# Overexpression of TP53INP2 Promotes Apoptosis in Clear Cell Renal Cell Cancer via Caspase-8/TRAF6 Signaling Pathway

**DOI:** 10.1155/2022/1260423

**Published:** 2022-05-14

**Authors:** Xunjia Li, Daixing Hu, Ying Li, Yan Luo, Bing Liang, Kexiao Yu, Weijian Xiong, Deyu Zuo

**Affiliations:** ^1^Department of Nephrology, Chongqing Traditional Chinese Medicine Hospital, Chongqing, China; ^2^Department of Endocrine and Breast Surgery, The Second Affiliated Hospital of Chongqing Medical University, Chongqing, China; ^3^Department of Pathology, College of Basic Medicine, Chongqing Medical University, Chongqing, China; ^4^Department of Orthopaedics, Chongqing Traditional Chinese Medicine Hospital, Chongqing, China; ^5^Department of Rehabilitation Medicine, Chongqing Traditional Chinese Medicine Hospital, Chongqing, China

## Abstract

Clear cell renal cell cancer (ccRCC) is a tumor of high malignancy, which can escape apoptosis. The tumor protein p53-inducible nuclear protein 2 (TP53INP2), known as an autophagy protein, is the essential part for autophagosome formation and sensitizes cells to apoptosis. Our study is aimed at exploring the role of TP53INP2 in ccRCC. We have identified the autophagy-related genes (ARGs) of differential expression in ccRCC patients with the help of the TCGA database by bioinformatics analysis. Our assays of quantitative real-time polymerase chain reaction (qRT-PCR) and western blot were for the determination on the both levels of mRNA and protein. Overexpression of TP53INP2 on cellular proliferation, migration, and apoptosis of ccRCC was verified in the ways of performing CCK-8, wound scrape, transwell and flow cytometry assays *in vitro*, and a mice tumor model *in vivo*. Transmission electron microscopy was used to measure autophagy formation. The underlying mechanisms of TP53INP2 on ccRCC were determined via coimmunoprecipitation. TP53INP2 was found highly associated with an outcome of worse overall survival (OS) in Kaplan-Meier curves, and this parameter in ccRCC tissues was also lower than the normal tissues. Overexpression of TP53INP2 inhibited ccRCC cellular proliferation, migration, and invasion, as well as the tumor growth of mice. Those cells treated with autophagy inhibitor chloroquine (CQ) or TP53INP2 increased the apoptosis rate. TP53INP2 promoted autophagy formation and elevated the ratio of LC3 II/LC3 I. However, TP53INP2 did not significantly decrease the p-mTOR level. In addition, TP53INP2 activates the expressions of caspase-3, caspase-8, and PARP. Caspase-8 and TNF receptor associated factor 6 (TRAF6) were found to bind to each other in the presence of TP53INP2. TP53INP2 induces apoptosis in ccRCC cells through caspase-8/TRAF6 pathway, rather than the autophagy-dependent pathway.

## 1. Introduction

Renal cell cancer (RCC) is a common urological malignancy, whose incidence was over an amount of 70,000 cases annually. It was in 2019 that there were about 14,000 patients died of RCC in America [1]. Clear cell renal cell cancer (ccRCC) is the major RCC subtype, whose patients made up for the majority of the diagnosed cases [2]. ccRCC is a high-risk vascular tumor with no clinical symptoms of manifestation at its early stage, and more than 25-30% of the patients were found along with cancer metastasis as soon as their initial diagnosis of ccRCC, resulted in patients' poor survival prognoses [3]. With the approval of tyrosine kinase inhibitors and rapamycin (mTOR) inhibitors, molecular targeted therapies have been shown to benefit the prognosis of ccRCC, improving overall survival (OS) by 30 months [4, 5]. However, such targeted therapies have drawbacks such as drug resistance and more severe side effects [5]. Despite of the improved diagnostic and therapeutic methods, its recurrence and metastasis might still happen in some ccRCC patients with high mortality rates [6]. Hence, any possible effort to dig out novel biomarkers would benefit the early detection and the treatment of patients with ccRCC [6]. Autophagy, type II programmed cell death, is a means of cellular homeostasis to maintain itself stable under conditions of starvation and hypoxia [7]. Previous study has revealed the role of autophagy in numerous biological processes, and it was also closely related to multiple diseases, including cardiomyopathy, neurodegenerative diseases, and cancers [8]. LC3 is an autophagosome membrane protein, regarded as a biomarker to estimate autophagy activity in mammalian cells, initially found in eukaryotic cells of higher animals [9]. A study found that in many RCC cell lines, 30-60% of the growing cells showed significant LC3-II spots, while in normal primary renal cell culture, this proportion was only 1-5% [10]. Importantly, an inhibition to autophagy in RCC leads to an improvement in the efficiency of many treatment strategies [10, 11]. A report of Zhang et al. also argued that the inhibition to autophagy would enhance the preferential toxicity of paclitaxel to renal cancer cells [11]. Chloroquine (CQ), an antimalarial drug that suppresses autophagy, makes these highly aggressive cells sensitive to the mTOR inhibitor tisirolimus [10]. Mammalian target of rapamycin (mTOR) is an essential molecule in the regulation to autophagy [12]. Activation of mTOR is a negative regulator in the process of autophagy, so the administration of mTOR inhibitors conducts an enhancement in both the inhibition to mTOR signaling pathway and the synergize cytotoxicity on RCC cells, thereby begetting the death of autophagic cells that modulate kinase-dependent proteins [13, 14]. However, the pathogenesis of autophagy in RCC remains unclear.

Apoptosis is a description to the programmed death of cells, on which the stability of the organism's internal environment greatly relies [15]. Apoptosis is closely associated with caspase activation. Caspases is a class of apoptotic protein which is categorized into two main groups, which are the initiator caspases (caspase-2, -8, -9, and -10) and the executioner caspases (caspase-3, -6, and -7) [15]. The complexity of the interaction amid autophagy and apoptosis was extensively studied so far [16]. An inhibition to autophagy might lead to the amassing of autophagosome membranes, functioning as the platforms for the formation of intracellular disc. Procystease-8 binds to the inner disk of phagocytes via ATG12-ATG5-FADD axis on the outer membrane or LC3-p62 on the inner membrane of autophagy [17, 18]. Ubiquitinated caspase-8's binding to p62 is accomplished with its ubiquitin binding domain, whereas its recruitment onto the autophagosome by binding to LC3-II is done with the LC3-interacting region (LIR) of p62 [17, 18].In tumor protein p53 inducible nuclear protein 2 (TP53INP2), the nuclear protein participates in the interaction with the nuclear hormone receptor, which travels from the nucleus to the cytoplasm and prompts the synthesis of proteins by a way of facilitating the ribosomal biogenesis in the nucleoli [19]. However, when nutrients are depleted, TP53INP2 comes to an interaction with the nuclear and deacetylated LC3 pools; subsequently, they are both rapidly transported into the cytoplasm to activate autophagy [19]. TP53INP2 positive regulatory role in the process of autophagy is established with its direct interaction with the LIR sequences of the whole Atg8 family members [20]. It was recently discovered that TP53INP2 is an ubiquitin binding protein that favors single and K63-linked ubiquitin chains with an ability to promote cellular apoptosis with the death receptor ligands [21]; besides that, it is also a trigger for caspase-8 activation [22]. TP53INP2' combination to caspase-8 and the ubiquitin ligase TRAF6 would lead to a promotion in a TRAF6-inducing ubiquitination and activation of caspase-8 [22].

In the present study, the gene expression microarray data was for the development in autophagy-related prognostic model, deemed as an independent OS index in ccRCC on the basis of the Cancer Genome Atlas (TCGA) database. We found that the TP53INP2's expression in ccRCC turned out a conspicuous lower level versus normal tissues, and it was proportional to the survival rate of patients with ccRCC, suggesting that TP53INP2 may be a tumor suppressor gene. However, the underlying mechanism of TP53INP2's role in ccRCC remains unclear. In summary, we proposed the following hypothesis: The autophagy-related gene TP53INP2 promotes the apoptosis of ccRCC cells by activating the caspase-8 apoptotic signaling pathway, functioning as an inhibitor in ccRCC. Both in vivo and in vitro experiments have verified that TP53INP2 inhibits ccRCC cells by regulating caspase-8 apoptotic signaling pathway, so as to provide theoretical basis for further understanding of TP53INP2 and drug therapy of ccRCC.

## 2. Materials and Methods

### 2.1. Autophagy-Related Genes and the Subjects

We retrieved a database from the Human Autophagy (HADb,http://www.autophagy.lu/index.html) for the identification of autophagy-related genes which are 234 in total. ccRCC-related RNA-seq data and clinical information were downloaded from the TCGA data portal (https://tcga-data.nci. http://nih.gov/tcga/), comprising the parameters of 539 ccRCC tissues and 72 adjacent nontumor tissues.

### 2.2. Procedures and Statistical Analysis

Our data analysis on the ARGs of differential expression within ccRCC and adjacent nontumor tissues was going along with our R package limma, with the thresholds of ∣log2 fold change (FC) | >1 and adjusted *P* value <0.05. Heatmaps of ARGs were performed with package “pheatmap” in R. Then, we commenced a Gene Ontology (GO) enrichment analysis with DAVID to distinguish the differentially expressed ARGs in the course of major biological processes (BPs), and in separate the cellular components (CCs) and the molecular functions (MFs). Our KEGG (Kyoto Encyclopedia of Genes and Genomes) enrichment analysis was for obtaining the signaling pathways where the ARGs of differential expression participate. We then had our univariate Cox and multivariate Cox proportional hazard regression analysis for the evaluation on the OS-related ARGs in ccRCC. Our prognostic model for OS was established on the basis of multivariate Cox proportional hazard regression analysis. In terms of the median value of the prognostic model, our subjects with ccRCC were grouped into the high-risk and the low-risk. Our Kaplan-Meier methods were built for the estimation on the OS between the groups of high-risk and low-risk. The R package of survival ROC was made for the creation of receiver operating characteristic (ROC) curves so as to assess the sensitivity and specificity of prognostic model. This section comes to an end with all our following bioinformatics analysis basing on the R software (version 3.3.1).

### 2.3. Cell Culture

We purchased the human kidney proximal tubular epithelial cell line (HK-2) cells from American Type Culture Collection (Manassas, VA, USA). Additionally, our embryonic kidney cells 293T as well as the human ccRCC cell lines 786-O, ACHN, and A498 were bought from Cell Bank, the Chinese Academy of Sciences (Shanghai, China). Those cells were grown in the standard DMEM containing 10% fetal bovine serum (FBS, Gibco, USA) under conventional culture conditions (5% CO_2_, 37°C).

### 2.4. Transfection

pCDNA3-FLAG-TP53INP2 and pCMV-FLAG-TRAF6 were the lentivirus vectors purchased from Shanghai Jima for our stably transfected ACHN cell line establishment. ACHN cells were grown in a six-well plate with the density of 2 × 10^5^ cells per well. Lipofectamine 2000 (Invitrogen) kit was utilized for transfection in accordance with the instructions. Sequences of siTRAF6 were shown as follows: sense 5′-AAGUGCUCAGUAGUCAGGACAUU-3′, antisense 5′-UGUCCUGACUACUGAGCACUUUU-3′; and siNC, 5′-UUCUCCGAACGUGUCACGUTT-3′. Then, the cells were selected with puromycin. Forty-eight hours after transfection with lentivirus vectors or siRNA, transfection efficiency was evaluated with our western blot and qRT-PCR.

### 2.5. RNA Extraction and RT-PCR

We extracted the total RNA from the cells following the TRIzol reagent instructions (Invitrogen, CA, USA). After that, we carried out reverse transcription with Promega reverse transcription kit (Promega, USA). A tube of 4 *μ*L RNA template was consumed in our reverse transcription reaction, which was performed on the platform of ABI9700 instrument (ABI, USA). Our reaction system was set up as follows: a 4 *μ*L of 5 × RT buffer, 0.4 *μ*L of random primers, 0.5 *μ*L of dNTPs (10 mM), 1 *μ*L of MMLV (U/*μ*L), 10.1 *μ*L of DEPC water, and 4 *μ*L of RNA template. Reaction conditions were defaulted at 37°C for 1 h and 95°C for 3 min. According to instructions of the Promega PCR mix, our reaction system was settled as follows: 10 *μ*L of PCR mix buffer2×, 1 *μ*L of upstream primer, 1 *μ*L of downstream primer, 4 *μ*L of template, and 6 *μ*L of DEPC water. Then, the reaction conditions were adjusted at 93°C for 3 min, following at 93°C for 30 s, and 55°C for 45 sec, 40 cycles in total, subsequently from 72°C for 5 min to 4°C lasting for standing by. Our primers' sequences were shown in Table 1.

### 2.6. Western Blotting

Cells were firstly scraped off, then they were well mixed with lysate on ice for 40 min; after that, the cells were transferred into a precooled EP tube with a pipette, then they were centrifuged in 14000 rpm at 4°C for 15 min. The concentrated proteins were determined with the BCA kit (Life-iLab, Shanghai, China). We had our electrophoresis condition at 100 V for 40 min and then altered it to 80 V for 1 h. Our membrane transfer condition was settled at 250 mA for 2 h. Those membranes were then commenced the 5% skimmed milk blocking at room temperature for 1 h. Primary antibody was added and rocked for 1 h at room temperature. After that, we had the membrane rinsed with PBST 3 times (10 min each time). Then, the membranes were added with secondary antibody, incubated at room temperature for 1 h. Finally, the membranes were washed with PBST twice. The sources of antibodies were the following: TP53INP2, p-mTOR, mTOR, LC3, p62, cleaved caspase-3, cleaved caspase-8, cleaved PARP (Cell Signaling Technology, Beverly, MA, USA), *β*-actin (SUNGENE BIOTECH, Tianjin, China), goat anti-rabbit IgG (HRP), and goat anti-mouse IgG (HRP) (Kangcheng, Shanghai, China).

### 2.7. Cell Proliferation Assay

Cell Counting Kit-8 (CCK-8) (Sigma, USA) was utilized for the assessment on the long-term cell survival, and every procedure was performed following the manufacturer's instructions. Cell suspensions were grown at 5 × 10^3^ cells per well in 96-well culture plates. We determined the cell viability with CCK-8 at a final concentration of 10% to each well with the absorbance at 450 nm by using a multiwell fluorescent plate reader (Thermo Scientific Varioskan Flash, Thermo Fisher Scientific, USA) every 24 h for 3 days. We plotted the proliferation curves in terms of the absorbance.

### 2.8. Wound Scrape Assay

Separately at 24 h and 48 h posttransfection, we grown those cells in 6-well plate and have them scratched with a 200 *μ*L pipette tip in the middle of the wells; then, they were cultivated with serum-free medium. Twenty-four hours after that, we made an estimation to the width of wounds in three-independent wound sites per group; then, we normalized the data to the control.

### 2.9. Transwell Migration Assay

786-O, ACHN, and A498 cells were inoculated into the transwell chamber of a 24-well plate (1 × 10 [5]/well) with 600 *μ*L of complete medium adding into the lower chamber. By observing the growth characteristics of the different cells, we took out the transwell chamber after 12–16 h cultivation. After 4% paraformaldehyde fixation, cotton swabs were to remove the remaining cells out of membrane surface. We finally performed a crystal violet staining assay and took the photographs with a microscope (MF53-N, Mingmei, China).

### 2.10. Cell Apoptosis Assay

Collected cells were centrifugated in 1000 r/min for 3 min, then washed twice with ice-cold PBS, gently resuspended in 500 *μ*L 1× annexin V binding buffer containing 5 *μ*L annexin V-FITC and 3 *μ*L of PI, and then incubated for 10 min at room temperature in dark. Apoptotic cell percentage was determined with flow cytometer (BD FACSCalibur, Becton-Dickinson, USA).

### 2.11. Cell Cycle Analysis

Procedures of cell cycle assay were shown as follow: cells from different groups were grown in 6-well plates and kept for 48 h. Then, we had them washed with ice-cold PBS and fixed them in 70% (*v*/*v*) ice-cold ethanol solution overnight at 4°C; these cells were analyzed in the following day with flow cytometry following the instruction of cell cycle analysis kit (Sigma, MO, USA). Information of cell cycle was determined with ModFit LT 4.0 software.

### 2.12. Subcutaneous Xenograft Model in BALB/c Nude Mice

The animal study was carried out in terms of the Ethics Committee of Chongqing Medical University. The male BALB/c nude mice aged 4–6 weeks were purchased from Chongqing Medical University and maintained in accordance with the institutional policies. Stable cell lines (ACHN/vector cells and ACHN/shRNA cells) were used. Details of the experiments performed are as described in our previous report [23]. Briefly, ACHN/vector cells or ACHN/shRNA cells were injected, and the tumor volumes were observed every 3 days and calculated. Tumors were surgically removed 30 days after injection and photographed. Tumor volume was assessed by caliper measurement and calculated according to the formula (*L* × *W* × *W*/2), where *L* represents length and *W* represents width.

### 2.13. Immunohistochemistry

Protein expressions of TP53INP2 (Cell Signaling Technology, Beverly, MA, USA) in tissues were assessed with immunohistochemistry. Formalin-fixed paraffin-embedded tissue sections were deparaffinized in xylene and rehydrated with graded ethanol, which were then boiled for 30 min in citrate buffer (10 mM, pH 6.0) for antigen retrieval. We made the endogenous peroxidase activity suppressed by exposing itself to 3% hydrogen peroxide for 10 min. Those slides were blocked with 5% BSA (Boster Bioengineering, Wuhan, China) and incubated with diluted primary antibodies at 4°C overnight. After an incubation with secondary antibody for 1 h at 37°C, those slides were visualized with 3,3′-diaminobenzidine (DAB) and counterstained with hematoxylin for microscopic examination. Terminal deoxynucleotidyl transferase-mediated dUTP-fluorescein nick end labeling (TUNEL) was conducted with the In Situ Cell Death Detection Kit, POD (11684817910, Roche, Switzerland).

### 2.14. Immunofluorescence

Twenty-four hours after transfection, the ACHN cells cultured on glass coverslips were fixed in 4% paraformaldehyde for 15 min and permeabilized with 0.01% Triton X-100 for 20 min at room temperature. After that, we had them blocked with 5% goat serum for 1 h at 37°C and performed an incubation with primary antibodies overnight at 4°C. The secondary antibodies were Alexa 488-conjugated goat anti-mouse or anti-rabbit IgG (Abcam, UK). Implementation of 4′,6-diamidino-2-phenylindole (Sigma-Aldrich) was for counterstaining the nuclei. Then, we took out fluorescent images with fluorescence microscope (IX70, Olympus, Japan). The primary antibodies were purchased from Abcam as follows: anti-LC3B (no. ab225383, 1 : 1000), anti-TRAF6 (no. ab40675, 1 : 500), anti-TP53INP2 (no. ab273012, 1 : 100), and caspase-8 (no. ab25901, 1 : 100).

### 2.15. Immunoprecipitation

ACHN cells were transfected with the plasmids ordered from the polyethyleneimine (PEI) of Polysciences Inc.; after the transfection, they were subjected to a 36-hour lysed with lysis buffer and underwent a pulling down assay with FLAG (Sigma) with all the procedures following the instructions. Immunocomplexes were done with SDS-PAGE, determined with western blot by using anti-FLAG, anti-TP53INP2, and anti-caspase-8 antibody.

### 2.16. Statistical Analysis

All data were analyzed by using GraphPad Prism 7 (San Diego, CA, USA) and shown in a way of the mean ± SD. Our statistical analysis was for the determination to the significance of the difference between the groups with one-way ANOVA and Turky test or Student's *t*-test. Outcomes with two-tailed and *P* values <0.05 were statistically significant.

## 3. Results

### 3.1. Functional Enrichment Analysis on the Autophagy-Related Genes of Differential Expression

**Figure 1:**
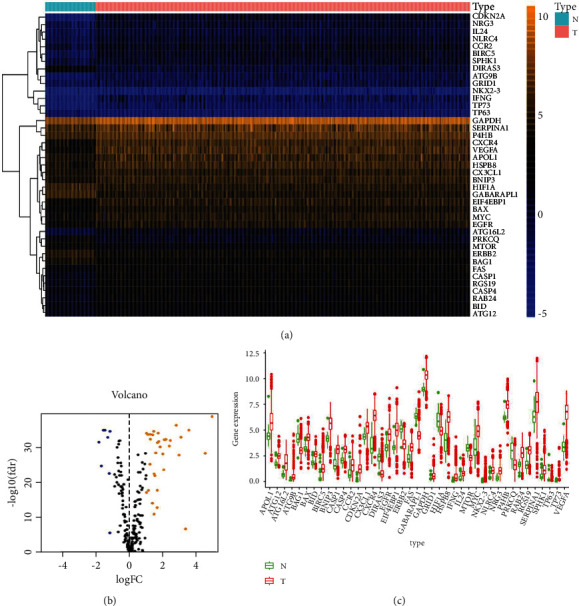
The autophagy-related genes of differential expression. (a) Heatmap of the autophagy-related genes of differential expression (ARG). N was tantamount to nontumor tissues; T was tantamount to tumor tissues. (b) Volcano plot on the ARGs of differential expressions. Orange dots indicated the ones of high expression whereas the blues were for the low expressions. (c) The boxplot for the ARGs of differential expression. Red and green regions separately represented tumor tissues and normal tissues.

Of all the 234 autophagy-related genes, we finally identified 41 differentially expressed ARGs, encompassing 33 genes of upregulation (CX3CL1, ATG12, BID, IL24, FAS, BAX, CASP4, CCR2, P4HB, GAPDH, GRID1, EGFR, MYC, BNIP3, SERPINA1, SPHK1, RAB24, RGS19, CASP1, NLRC4, NRG3, APOL1, EIF4EBP1, HSPB8, ATG16L2, BIRC5, CXCR4, ATG9B, TP73, NKX2-3, VEGFA, IFNG, and CDKN2A) and 8 downregulated ARGs (DIRAS3, PRKCQ, GABARAPL1, ERBB2, BAG1, HIF1A, TP63, and mTOR) (Figure 1(a)). Volcano plot and box plot revealed the differentially expressed ARGs' expression patterns within tumor and nontumor tissues (Figures 1(b) and 1(c)).

GO enrichment analysis was performed according to the differentially expressed ARGs. The top differentially expressed ARG-associated biological processes included regulation of endopeptidase activity, regulation of peptidase activity, autophagy, and process utilizing autophagic mechanism. Supplementary Figure 1(a) also showed the top cellular components including autophagosome and cytosolic part and top molecular functions including ubiquitin protein ligase binding, ubiquitin-like protein ligase binding, and cytokine receptor binding. In the KEGG pathway enrichment analysis, differentially expressed ARGs were shown to be notably associated with human cytomegalovirus infection, shigellosis, and HIF-1 signaling pathway (Supplementary Figure 1(B)-1(D)).

### 3.2. The Identification into Prognosis-Related ARGs and the Construction on Prognosis-Related Prediction Model

The univariate Cox regression analysis was for the identification on OS-related ARGs (Table 2). Our multivariate Cox regression analysis identified seventeen genes, including BID, ATG4B, CASP4, ZFYVE1, PRKAR1A, CAPN10, NFKB1, NPC1, TP53INP2, ULK1, MAPK1, HSPA8, EIF2S1, CDKN2A, PTEN, BAG1, and BNIP3, which were deemed as the OS independent prognostic indicators; and we selected them as the developing prognostic signature (Table 3). OS − related prediction model = (0.632∗the expression value for BID) + (1.234∗the expression value for ATG4B) + (0.368∗the expression value for CASP4) + (−0.453∗the expression value for ZFYVE1) + (0.911∗the expression value for PRKAR1A) + (−0.753∗the expression value for CAPN10) + (−0.720∗the expression value for NFKB1) + (0.395∗the expression value for NPC1) + (−0.477∗the expression value for TP53INP2) + (0.368∗the expression value for ULK1) + (0.470∗the expression value for MAPK1) + (−0.500∗the expression value for HSPA8) + (0.883∗the expression value for EIF2S1) + (−0.228∗the expression value for CDKN2A) + (−0.722∗the expression value for PTEN) + (−0.611∗the expression value for BAG1) + (−0.359∗the expression value for BNIP3).

**Figure 2:**
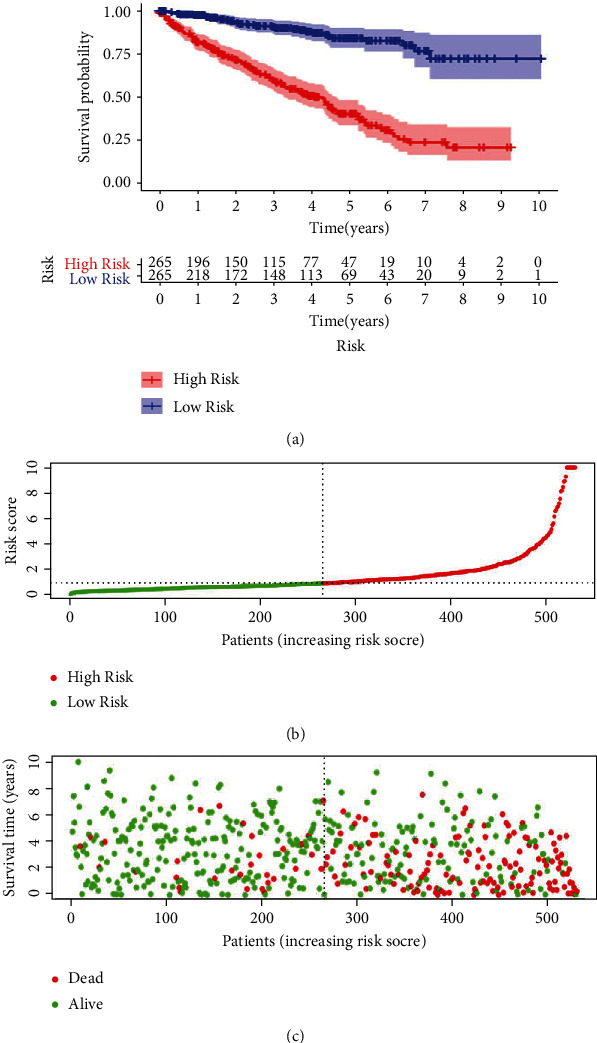
The autophagy-related prognostic model on the patients of ccRCC. (a) The Kaplan-Meier curve revealed a fact that the high-risk group came in a shorter OS value than the low-risk one. (b) Prognostic model distribution on the patients of ccRCC. (c) OS value of patients in the TCGA dataset. The region of red color indicated a higher risk score whereas the green color meant lower risk.

We made a division to the 539 ccRCC cases into high- and low-risk groups basing on the median values of our OS-related prediction model. Kaplan–Meier survival curves showed that the high-risk group had a higher mortality rate than the low-risk group (*P* < 0.001) (Figure 2(a)). The distribution of the seventeen-ARG risk score and the relapse status of the ccRCC patients and the ARG expression signature were shown in Figures 2(b) and 2(c).

In terms of our median value of the seventeen genes in prediction model, high levels of BID, ATG4B, CASP4, CAPN10, and ULK1 were conspicuously correlated with the worse overall survival in Kaplan–Meier curves (Supplementary Figure 2). On the contrary, low levels of ZFYVE1, PRKAR1A, NFKB1, TP53INP2, MAPK1, HSPA8, BAG1, EIF2S1, PTEN, and BNIP3 were in a significant association with the worse overall survival in our Kaplan–Meier curves (Supplementary Figure 3). To our surprise, the expression of NPC1 and CDKN2A showed no significant correlation with OS in Kaplan–Meier curves (Supplementary Figure 4).

### 3.3. Autophagy-Related Signature is an Independent Factor for ccRCC Prognosis

**Figure 3:**
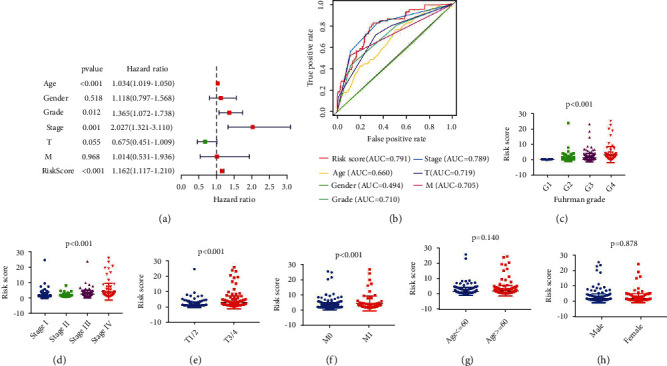
Autophagy-related signature was regarded as an independent factor for ccRCC diagnosis. (a) Multivariate Cox regression analyses were for the verification to the independent value on autophagy-related manifestation, so that the OS would be evaluated. (b) A ROC analysis on the OS for the manifestation and the clinicopathologic parameters. (c)–(h) Clinicopathological significance of the OS-related prognostic ccRCC model.

Our univariate Cox analysis revealed that age, AJCC stage, Fuhrman grade, T status, M status, and the autophagy-related signature were all the associated factors to the OS of ccRCC patients (Table 4). Moreover, our multivariate Cox regression analysis was performed for the verification on the independent predictive value of clinicopathological parameters and autophagy-related signature for OS. Age, AJCC stage, Fuhrman grade and the autophagy-related signature were the independent predictive factors for the overall survival in ccRCC patients (Figure 3(a)). ROC curves shown in Figure 3(b) were the demonstration on clinicopathological parameters and autophagy-related signature for OS. The autophagy-related signature was the areas under the curve (AUC) in ROC curve with the value of 0.791. Of all the 539 ccRCC patients, there were large amounts of them scant of N status information, making our subsequent analysis on relationship amid N status and OS ambiguous.

The OS-related prediction model values of Fuhman grade were higher than those of the low grade (*P* < 0.001), and those of AJCC stage were higher than the low AJCC stage (*P* < 0.001); those values of T3/4 were also higher than the T1/2 (*P* < 0.001), and those of M1 are higher than the M0 (*P* < 0.001). There was no signal distinction amid the values of OS-related prediction model between patients aged >60 and patients aged ≤60 (*P* = 0.140), so was that of male and female (*P* = 0.878) (Figures 3(c)–3(h)).

### 3.4. Integrated Analysis on ccRCC Unveils TP53INP2's Possible Biomarker Role in ccRCC

**Figure 4:**
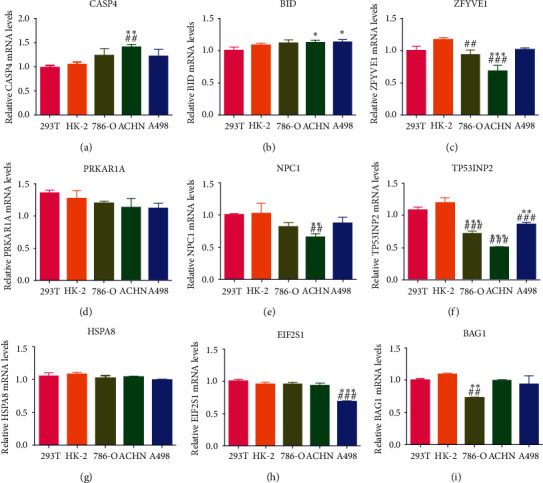
The relative mRNA level of autophagy-related genes BID (a), CASP4 (b), ZFYVE1 (c), PRKAR1A (d), NPC1 (e), TP53INP2 (f), HSPA8 (g), EIF2S1 (h), and BAG1 (i) in ccRCC cell lines was detected by qRT-PCR. Data were shown as mean ± SD of three or four independent experiments. Compared to the 293T group: ^∗^*P* < 0.05; ^∗∗^*P* < 0.01; ^∗∗∗^*P* < 0.001; compared to the HK-2 group: ^##^*P* < 0.01, ^###^*P* < 0.001.

**Figure 5:**
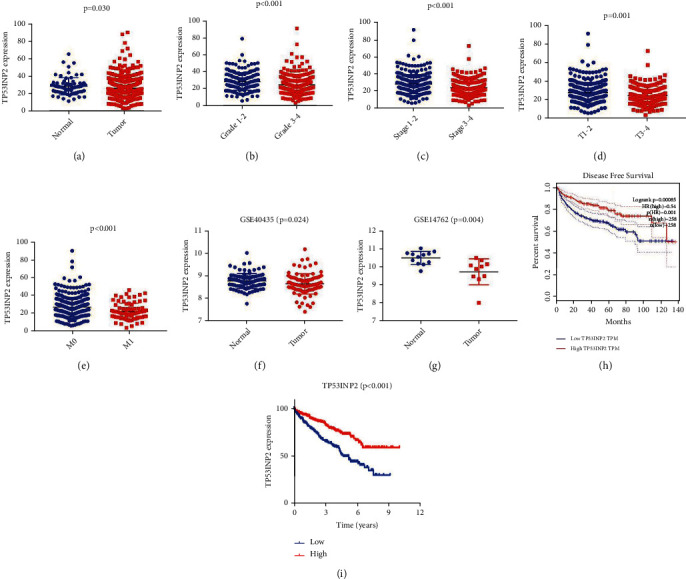
TP53INP2 expression and ccRCC patients' clinical data from the TCGA dataset and the GSEA analysis. The TP53INP2 expression in ccRCC tissues (a), Fuhman grade (b), American Joint Committee on Cancer (AJCC) stage (c), TNM status (d) and (e) based on TCGA dataset. (f) and (g) The TP53INP2 expression in ccRCC tissues based on GSEA analysis. (h) and (i) Low expression of TP53INP2 was associated with worse overall survival in Kaplan-Meier curves.

Of the 17 ARGs we previously verified, genes BID, CASP4, ZFYVE1, PRKAR1A, NPC1, TP53INP2, HSPA8, EIF2S1, and BAG1 were detected in 293T, HK2, 786-O, ACHN, and A498 cell lines and they were all rarely studied in renal cell carcinoma. Our results showed that the mRNA expression of TP53INP2 was significantly lower in ccRCC cells when compared with the normal renal cells, and that difference turned the most remarkable versus other genes (Figures 4(a)–4(i)). We then combined the data of TP53INP2 expression and a clinical information from the TCGA dataset. To begin with our assay, we shut out the patients with incomplete clinical information. Then, the outcomes showed that the TP53INP2 expression of cancer tissues turned a significant low level than the normal tissues (*P* < 0.05), and that in high Fuhman, grade was also lower than the low grade (*P* < 0.001); the TP53INP2 expression of cancer tissues in high AJCC stage was lower than the low AJCC stage (*P* < 0.001), and it was also lower in the T3/4 when compared with the T1/2 (*P* = 0.001), and the TP53INP2 expression in M1 was lower than the M0 (*P* < 0.001) (Figures 5(a)–5(e)). Meanwhile, based on the GSEA analysis (GSE40435 and GSE14762), TP53INP2 expression of the cancer tissues was lower than the normal tissues (Figures 5(f) and 5(g)). Moreover, TP53INP2 showed significantly correlated to worse overall survival in Kaplan-Meier curves (Figures 5(h) and 5(i)). All the results above revealed that TP53INP2 plays a possible biomarker role in ccRCC.

### 3.5. TP53INP2 Inhibited the Proliferation of ccRCC Cell Lines

**Figure 6:**
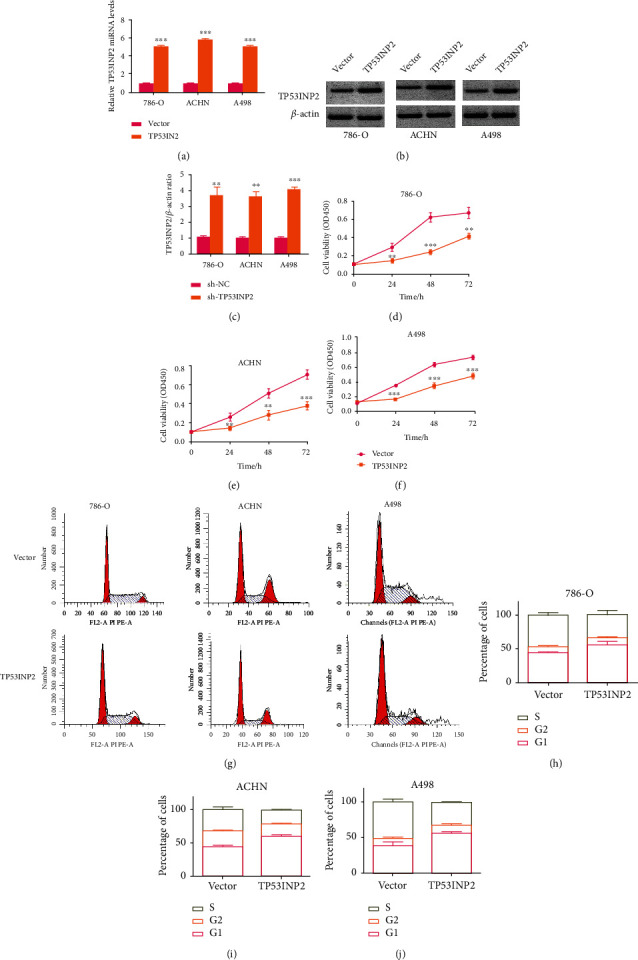
Overexpression of TP53INP2 resulted in an inhibition to cellular proliferation in ccRCC cell lines. (a) TP53INP2 overexpression was stably transfected into the 786-O, ACHN, and A498 ccRCC cell lines. The relative mRNA level of TP53INP2 was determined with qRT-PCR. (b) and (c) The level of TP53INP2 protein was determined with western blot. Relative values were estimated with Image J. (d)–(f) CCK-8 assay was made for the detection on the cell viability of 786-O, ACHN, and A498. (g)–(j) The flow cytometry was performed for the detection on the cell viability of 786-O, ACHN, and A498. Data were presented as mean ± SD of three or four independent experiments. ^∗∗^*P* < 0.01; ^∗∗∗^*P* < 0.001.

TP53INP2's biomarker role in ccRCC cells has been revealed in last section, and we would manage to dig out its biological role in 786-O, ACHN, and A498 cell lines by overexpressing this gene, respectively. qRT-PCR and western blot unveiled an outcome that TP53INP2 was highly expressed in all these cell lines on both mRNA and protein level (Figures 6(a)–6(c)). We had an overexpression of TP53INP2 in 786-O, ACHN, and A498 cell lines separately, and our CCK8 assays showed that the cell viability of these cell lines was all remarkably suppressed (Figures 6(d)–6(f)). Our subsequent flow cytometry assay revealed that all the cell lines manifested a notable decreasing proportion in phase S whereas turned an increasing proportion in phase G1 (Figures 6(g)–6(j)). And these results implied that all the ccRCC cell lines were arrested in phase G1, resulting in reduced cell proliferation.

### 3.6. TP53INP2 Inhibited Invasion and Promoted Apoptosis in ccRCC Cell Lines

**Figure 7:**
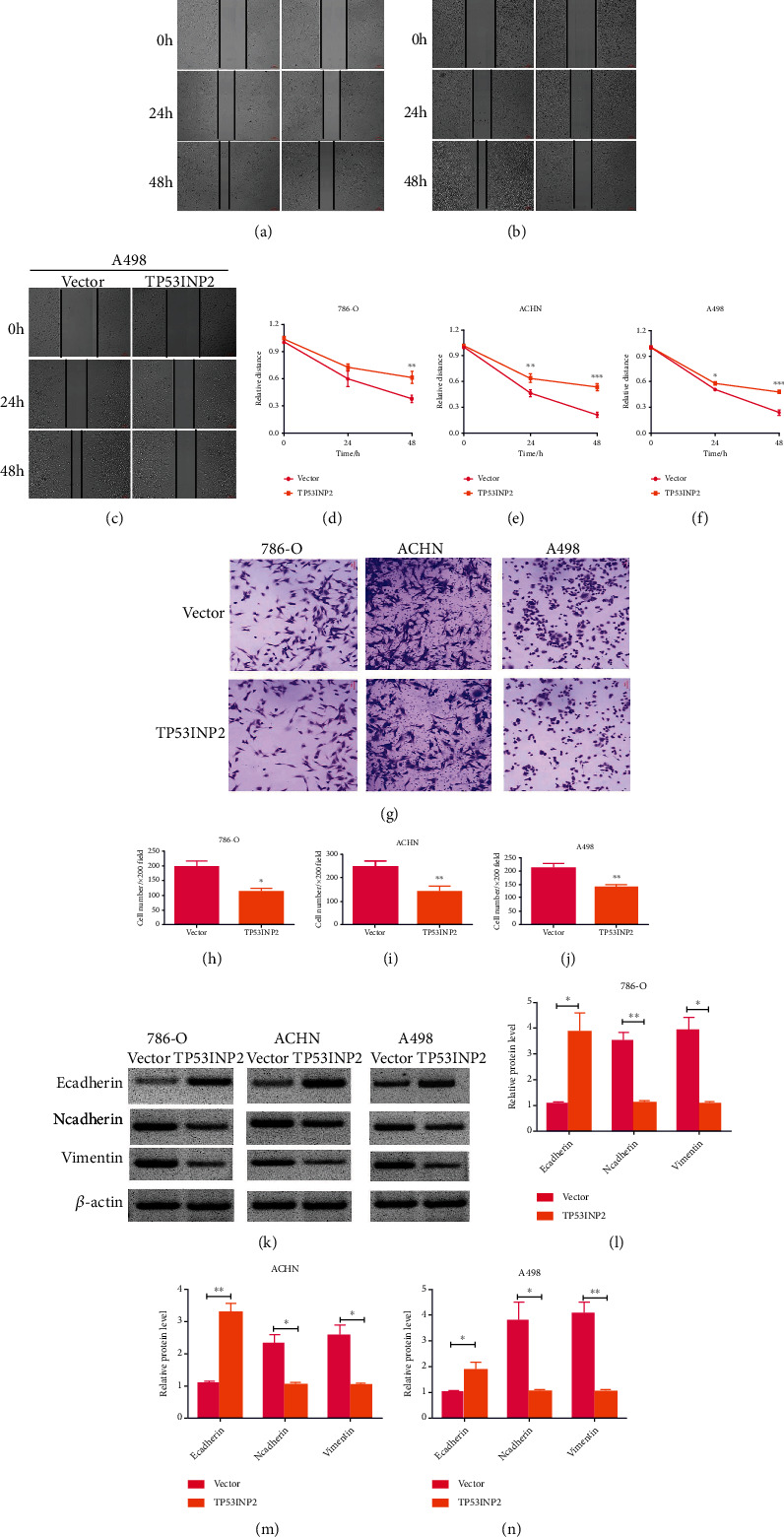
Overexpression of TP53INP2 led to inhibitions on the cellular migration and the cellular invasion of ccRCC cell lines. Both wound healing experiment (a)–(f) and transwell assays (g)–(j) were for the verification on the efficacy of TP53INP2 overexpression on migrative and invasive capabilities of 786-O, ACHN, and A498 cell. (k)–(n) Levels of epithelial marker E-cadherin and mesenchymal marker (Ncadherin and vimentin) in TP53INP2-overexpressed cells were measured with western blot. Data were presented as mean ± SD of three or four independent experiments. ^∗^*P* < 0.05; ^∗∗^*P* < 0.01; ^∗∗∗^*P* < 0.001.

We would have transwell and the scratch wound assays in this section to verify TP53INP2's effects on both the cellular invasion and migration of ccRCC. The scratch wound assay showed that overexpression of TP53INP2 disabled the migration of ccRCC cells after being scratched (Figures 7(a)–7(f)). Moreover, transwell assay showed that TP53INP2 significantly alleviated invasion in the 786-O, ACHN, and A498 cells (Figures 7(g)–7(j)).

**Figure 8:**
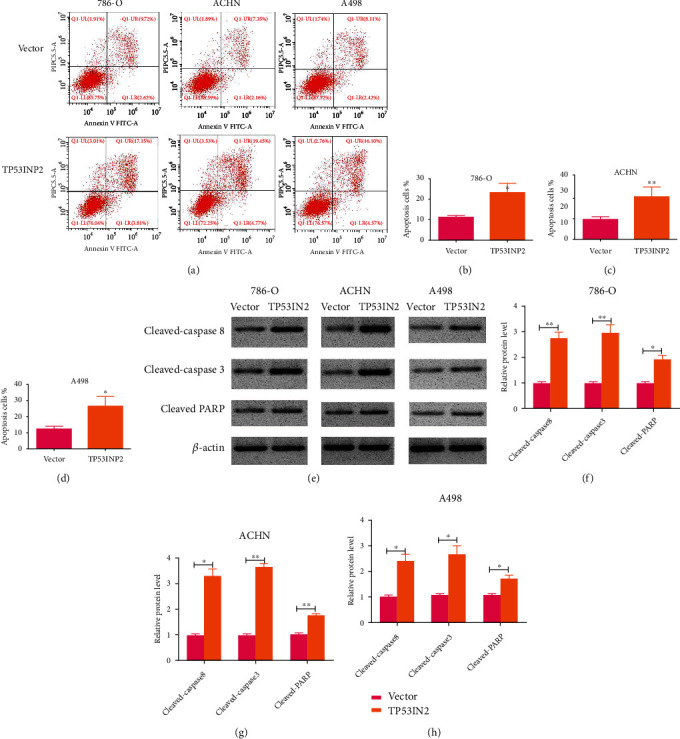
Overexpression of TP53INP2 promoted cell apoptosis in ccRCC cell lines. (a)–(d) Apoptosis rates of 786-O, ACHN, and A498 cell were detected by flow cytometry after overexpressing TP53INP2. (e)–(h) Levels of proapoptotic protein cleaved caspase-3, cleaved caspase-8, and cleaved PARP were measured by a way of western blot. Data are presented as mean ± SD of three or four independent experiments. ^∗^*P* < 0.05; ^∗∗^*P* < 0.01.

Simultaneously, we detected the expressions of invasion-related proteins in these cells. Our western blot assay showed that TP53INP2-overexpressed ccRCC cells come in an increasing levels of epithelial marker E-cadherin whereas resulted in a decrease in mesenchymal marker expression (Ncadherin and vimentin) (Figures 7(k)–7(n)). Then, flow cytometry showed that TP53INP2-overexpressed ccRCC cells turned a higher percentage of cell apoptosis in contrast to the control (Figures 8(a)–8(d)). The expressions of proapoptotic proteins cleaved caspase-3, cleaved caspase-8, and cleaved PARP were markedly upregulated in the TP53INP2-overexpressed ccRCC cell lines when compared with our control group (Figures 8(e)–8(h)).

### 3.7. TP53INP2 Accelerated Autophagy and Apoptosis in ACHN Cells

**Figure 9:**
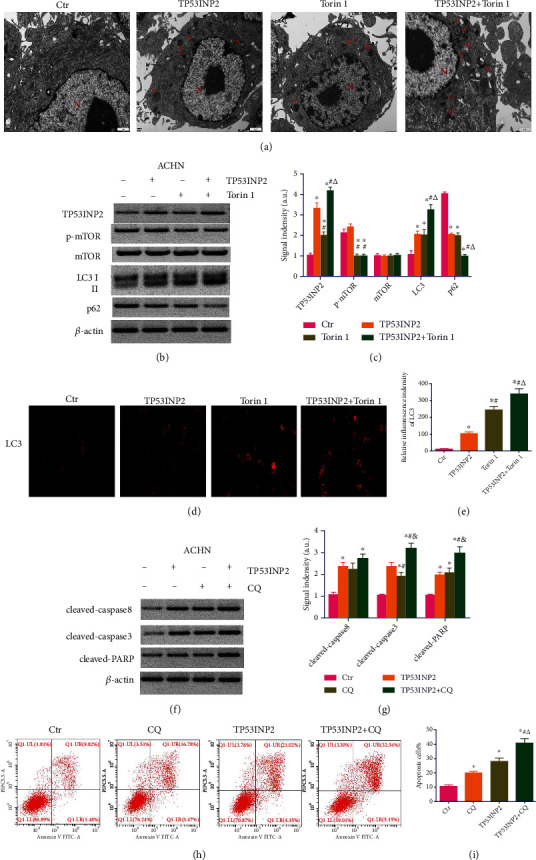
Overexpression of TP53INP2 induces apoptosis in ACHN cells through a nonautophagy dependent pathway. (a) The number of autophagic vacuoles in ACHN cells treated with TP53INP2, mTOR inhibitor Torin1, and TP53INP2+Torin1. (b) and (c) The levels of autophagy-related proteins (TP53INP2, p-mTOR, mTOR, LC3, and p62) were evaluated with western blot. (d) and (e) The expression intensity of LC3 was estimated with immunofluorescence. (f) and (g) The levels of proapoptotic protein in ACHN cells treated with TP53INP2, autophagy inhibitor chloroquine (CQ), and TP53INP2+CQ. (h) and (i) Flow cytometry assay was performed for the detection on the apoptosis rate between different groups. Data are presented as mean ± SD of three or four independent experiments. ^∗^*P* < 0.05, compared to control; ^#^*P* < 0.05, compared to TP53INP2; ^△^*P* < 0.05, compared to Torin1 in (c) and (e), compared to CQ in (i), and *P* < 0.05, compared to CQ in G.

Microscopically speaking, administration of TP53INP2 or mTOR inhibitor Torin1 might lead to an increasement in autophagic vacuoles' number in ACHN cells. Moreover, amount of autophagosomes in the cells of TP53INP2+Torin1 was more than that of merely TP53INP2 or Torin1 (Figure 9(a)). Western blot assay revealed that the overexpression of TP53INP2 in ACHN cells come into no significant effect on mTOR phosphorylation, while the phosphorylation level of mTOR was decreased in Torin1-treated ACHN cells. In contrast to the control, both western blot and immunofluorescence assays turned out a significant increase in LC3 levels in the TP53INP2-overexpressed or Torin1-treated ACHN cells, as well as an enhancement in cells treated with TP53INP2+Torin1 (Figures 9(b)–9(e)). The expression trend of p62 was opposite to LC3 (Figures 9(b)–9(c)). It suggests that TP53INP2 can activate autophagy. Since autophagy and apoptosis are closely related, we added the autophagy inhibitor chloroquine (CQ) to investigate whether TP53INP2 induces cell apoptosis through the activation of autophagy. Western blot assay showed that merely CQ or TP53INP2 might elevate the expressions of proapoptotic proteins (cleaved caspase-3, cleaved caspase-8, and cleaved PARP) versus the control group. Furthermore, the expressions of proapoptotic proteins were signally elevated in TP53INP2+CQ group when compared with the TP53INP2 group (Figures 9(f) and 9(g)). The flow cytometry exhibited that the cell apoptosis was markedly enhanced in CQ-, TP53INP2-, and CQ+TP53INP2-treated ACHN cells, and the apoptosis rate in cells treated with CQ+TP53INP2 was the highest (Figures 9(h) and 9(i)). These results demonstrated that the TP53INP2 activated autophagy is a protective effect on ACHN, and the proapoptotic effect of TP53INP2 on ACHN is independent of the autophagy pathway.

### 3.8. Overexpression TP53INP2 Inhibits Tumor Growth In Vivo

**Figure 10:**
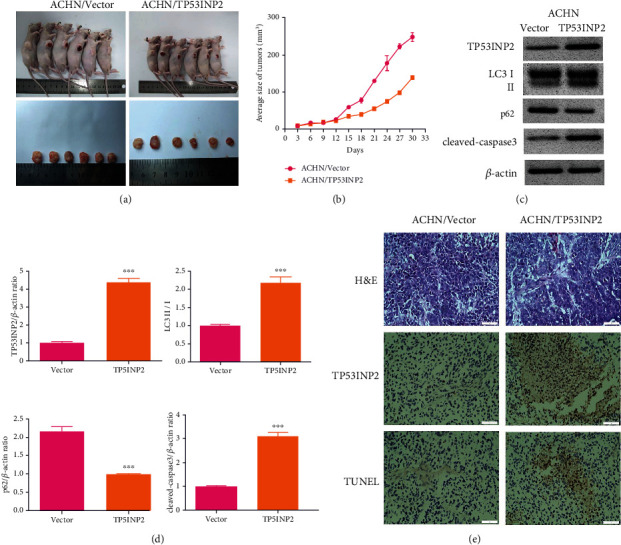
Overexpression of TP53INP2 inhibits tumor growth in mice. (a) and (b) Tumorous size was determined every three days. TP53INP2- or empty vector-transfected ACHN cells were injected into mice (*n* = 6). (c) and (d) The levels of autophagy-related and proapoptotic protein in tumor tissue were determined by a way of western blot. (e) Representative H&E staining images of tumor tissue (400x). TP53INP2 expression was detected with immunohistochemistry (400x). TUNEL was used to detect apoptosis in tumor tissues (400x). ^∗∗∗^*P* < 0.001.

Our further experiments would be conducted in animals for the detection on TP53INP2's effects on the tumor evolution. TP53INP2- or empty vector-transfected ACHN cells were injected into nude mice. After overexpressing TP53INP2 in vivo, the size of the tumor was significantly reduced (Figures 10(a) and 10(b)). Western blot showed that the LC3 II/I ratio and cleaved caspase-3 level of mice transfected with TP53INP2 were greatly higher than those of the control, while the p62 level was lower versus the control (Figures 10(c) and 10(d)). In addition, the experimental results showed that the TP53INP2 was successfully overexpressed in mice (Figures 10(c)–10(e)). The H&E staining images showed that the cell morphology was normal in the control group, whereas the number of tumor cells decreased and cell borders were indistinct in the TP53INP2. TUNEL assay revealed that the overexpression of TP53INP2 promoted apoptosis of tumor cells (Figure 10(e)).

### 3.9. TP53INP2 Promoted Cell Apoptosis by Regulating Caspase-8/TRAF6 Signaling Pathway

**Figure 11:**
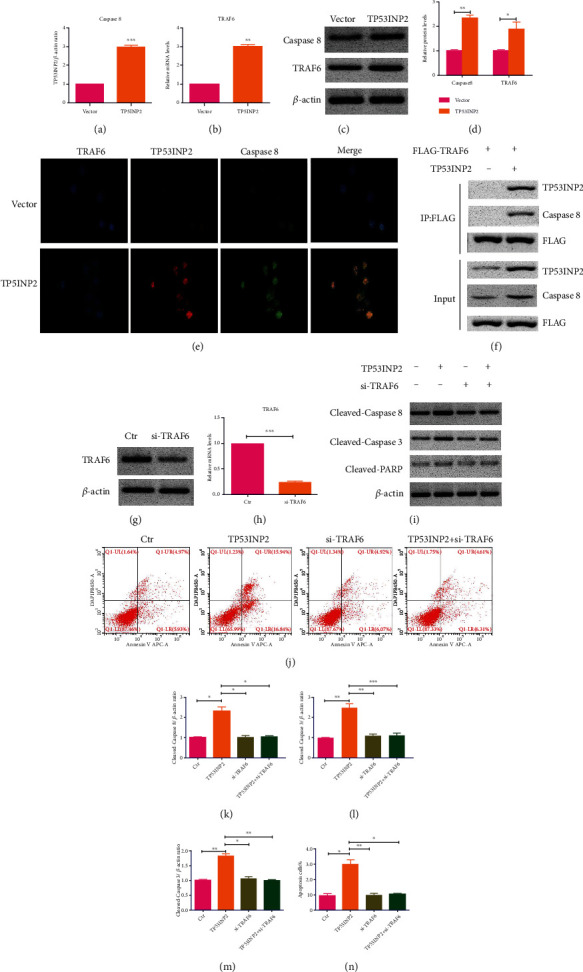
Overexpression of TP53INP2 promoted cell apoptosis via caspase-8/TRAF6 pathway in ACHN cells. (a) and (b) Both mRNA levels of caspase-8 and TRAF6 were detected with qRT-PCR in TP53INP2-treated ACHN cells. (c) and (d) The protein levels of both caspase-8 and TRAF6 were evaluated with western blot. (e) Overexpression of TP53INP2 upregulated both expressions of caspase-8 and TRAF6, which was verified with immunofluorescence. (f) ACHN cells expressing FLAG-TRAF6 and TP53INP2 were immunoprecipitated with FLAG resin, and immunocomplexes were subjected to western blot analysis with anti-FLAG, anti-TP53INP2, and anti-caspase-8 antibody. (g) and (h) TRAF6 silence was transiently transfected into the ACHN cell lines. The relative mRNA level and protein level of TRAF6 was estimated with qRT-PCR and western blot. (i) and (k)–(m) Protein levels of cleaved caspase-3, cleaved caspase-8, and cleaved PARP were measured in cells administrated with TP53INP2, siTRAF6, and TP53INP2+siTRAF6. (j) and (n) Flow cytometry was accomplished for the detection on the apoptosis rate in different groups. Data were presented as mean ± SD of three or four independent experiments. ^∗^*P* < 0.05; ^∗∗^*P* < 0.01; ^∗∗^*P* < 0.001.

Our exploration on the regulatory mechanism of TP53INP2 to ccRCC cell apoptosis was enforced, the proteins binding to TP53INP2 were obtained by co-IP assay, and the proteins directly binding to TP53INP2 were detected by western blot assay. TRAF6 was reported to add K63 chains to caspase-8 when an apoptosis was induced by death receptor. The caspase-8, TRAF6, and TP53INP2 are the components on a same complex; besides that, TP53INP2 works as a scaffold for TRAF6-induced caspase-8 ubiquitination [22]. Therefore, the expressions of TRAF6 and cleaved caspase-8 in TP53INP2-treated ACHN cells were determined by qRT-PCR, western blot, and immunofluorescence. And the results turned out that the TP53INP2 upregulated both the expressions of cleaved caspase-8 and TRAF6 when they were compared with the control (Figures 11(a)–11(e)). The interaction between cleaved caspase-8 and TRAF6 was observed in the presence of TP53INP2 by co-IP assay, thus suggesting a fact that cleaved caspase-8, TRAF6, and TP53INP2 are on the same complex (Figure 11(f)). Subsequently, we silenced TRAF6 in ACHN cells (Figures 11(g) and 11(h)). We found that the absence of TRAF6 resulted in an impaired increasement of proapoptotic proteins (cleaved caspase-3, cleaved caspase-8, and cleaved PARP) and apoptosis rates induced by the overexpression of TP53INP2 (Figures 11(i)–11(n)). Collectively, our results suggested that the TP53INP2 inhibited renal cell carcinoma in a way of regulating the caspase-8/TRAF6 apoptotic signaling pathway.

## 4. Discussion

Autophagy is an essential procedure in the biological function accomplishment of ccRCC [24]. The autophagy-related genes, signaling pathways, and markers are associated with ccRCC, and to further analyze the inner link between autophagy molecules to clarify conversion between autophagy and apoptosis, they can provide new ways for an early diagnosis on ccRCC and provide novel strategy to clinical treatment [10, 24, 25]. In this study, large-scale gene expression profile was paged for the investigation on the association within autophagy-related genes and clinicopathological characteristics, as well as OS in ccRCC. Autophagy-related genes (ARGs) of differential expression were identified in ccRCC patients with the help of the TCGA database. A univariate Cox regression model was built for the analysis on the expression level of ARGs and overall survival for the purpose of selecting autophagy-related prognostic genes. Then, we developed a novel prognostic model on the basis of 17 ARGs (BID, ATG4B, CASP4, ZFYVE1, PRKAR1A, CAPN10, NFKB1, NPC1, TP53INP2, ULK1, MAPK1, HSPA8, EIF2S1, CDKN2A, PTEN, BAG1, and BNIP3), all of which together could be the independent prognostic indicators for ccRCC patients. Of those genes, ATG4B, CAPN10, NFKB1, ULK1, MAPK1, CDKN2A, PTEN, and BNIP3 were reported to be the closely associated factors in renal cancer development. ATG4B was involved in the progression-free survival of ccRCC patients of pazopanib treatment [26]. CAPN10 has an inseparable relationship with renal function [27]. The promoter polymorphism of NFKB1 is known as an increased risk of RCC [28]. High expression level of ULK1 has been observed and closely related to poor survival in renal cancer [29]. Wei et al. found that MAPK1 is a vital gene in the VHL-HIF1*α* pathway in RCC patients [30]. Previous research has shown that the inactivation of CDKN2A is associated with the occurrence and development of ccRCC [31]. Lee et al. reported that low expression of PTEN tended to correlate with poor progression-free survival of RCC patients treated with sunitinib [32]. Restoration of BNIP3 expression would lead to inhibition to growth and a promotion in apoptotic for RCC cell lines [33]. However, the functions of BID, CASP4, ZFYVE1, PRKAR1A, NPC1, TP53INP2, HSPA8, EIF2S1, and BAG1 gene have not been reported in RCC, suggesting that the validation at cellular level, as well as cellular level studies, may reveal the function of these genes in both RCC development and progression.

Our analysis results showed that TP53INP2 was signally associated with worse overall survival (OS) in Kaplan-Meier curves. Moreover, TP53INP2 was hardly expressed in ccRCC tissues when compared with normal tissues. The above results suggest that TP53INP2 is indispensable for ccRCC development. TP53INP2 has not only the function on tumor inhibition but also on the ability to inhibit tumor metastasis, which was consistent with our results [34, 35]. Screening to cancer cell lines also implied that those cells with higher protein expressions of TP53INP2 are more prone to being the ones of receptor-induced apoptosis. Expression of TP53INP2 might be deemed as the biomarker in predicting human liposarcoma malignancies [36]. In bladder cancer, TP53INP2 is a modulator in cell migration, invasion, and EMT by going with the GSK-3beta/beta-catenin/Snail1 pathway [34]. Low expression of TP53INP2 in neck squamous cell carcinoma is known as a poor prognosis for an intervention in TP53INP2 expression that would lead to an advancement in cell proliferation [35]. Our results also showed that TP53INP2 has tumor inhibition function. Overexpressed TP53INP2 is found to suppress the activity, migration, and invasion of ccRCC cells, thereby inhibiting tumor growth in mice and promoting cell apoptosis.

Autophagy is an evolutionarily conserved biological progression, where the cytoplasmic materials and the organelles are sequestered into autophagosomes, and those molecules are further disintegrated in the autolysosomes so that the material cycle and the cellular homeostasis would be maintained [37]. On the basis of current studies, the importance of autophagy to tumorous biological development is well witnessed [38–40]. TP53INP2 involves in basic autophagy by the way of modulating the autophagosome formation [21]. After the autophagy is triggered, TP53INP2 would be relocated out of nucleus to autophagic vacuoles so as to facilitate recruit LC3 the autophagy mediator into these structures [41]. Overexpression of TP53INP2 in human liposarcoma cells would lead to an increasement in LC3 II/LC3 I ratio as well as a decreasement in p62 expression [36]. We observed in the present study that merely TP53INP2 or mTOR inhibitor Torin1 could initiate the autophagy in ACHN cell lines. Parameter of the LC3 II/LC3 I ratio is proved to be an indicator of autophagy, which was significantly increased in this context. The positive expression rate of LC3 in renal cancer tissues was shown evidently lower than the adjacent tissues, and the expression of LC3 was correlated with renal cancer development. Moreover, treatment with autophagy inhibitor CQ or TP53INP2 alone will increase the apoptosis rate of ccRCC cells. Aberrant AKT/mTOR pathway activation is broadly seen in various malignant tumors with a consequence of accelerating cellular proliferation, increasing cancerous resistance to apoptosis and advancing the invasion and metastasis of tumors [42]. MTOR is capable of tremendously affecting autophagy process, so the AKT/mTOR signaling pathway is able to negatively manipulate autophagy, but an intervention in the activation of mTOR would beef up autophagy [43, 44]. However, our results showed that the TP53INP2 overexpression did not efficiently downregulate mTOR phosphorylation. This finding suggests that TP53INP2-induced autophagy is not dependent on mTOR signaling pathway. TP53INP2 induces apoptosis in ccRCC cells through a nonautophagy dependent pathway.

Autophagy presents a complex and contradictory process in each stage of tumor. On the one hand, basic autophagy could slow down normal-cell transformation; on the other hand, under the condition of long-term metabolic damage, oxidative stress and lack of nutrients and persistent and excessive upregulation of autophagy would also cause cellular death [45]. Growing clinical evidence have been done and found that both autophagy enhancers and inhibitors may induce tumor cell apoptosis [46–48]. Caspase is the main executor of apoptosis, caspase-8 initiates apoptosis through autoactivation, and caspase-3 breaks down cellular proteins and promotes apoptosis [15, 49]. TP53INP2 is a member of those dual-function proteins to initiate autophagy or apoptosis in different conditions. In this study, we found that the TP53INP2 and autophagy inhibitor treatment can activate caspase-3, caspase-8, and PARP and further promote ccRCC cells into apoptosis. TP53INP2 interplays and initiates caspase-8 in a way of facilitating its TRAF6-inducing ubiquitination, thus shifting the response on cell death of apoptosis [22]. Silencing TRAF6 alone did not significantly promote the expressions of cleaved caspase-3, cleaved caspase-8, and cleaved PARP and apoptosis in this study. The apoptotic rate of cells treated with both overexpression of TP53INP2 and silencing of TRAF6 decreased compared with those treated with the overexpression of TP53INP2. Our experiment further confirms this caspase-8 and TRAF6 bind to each other in the presence of TP53INP2. These results imply that TP53INP2 inhibited renal cell carcinoma by regulating caspase-8 apoptotic pathway.

All the results demonstrated that the inhibitory effects of autophagy-related gene TP53INP2 work out in tumor growth in both vitro and vivo. Moreover, TP53INP2 induces apoptosis in ccRCC cells through a nonautophagy dependent pathway, suggesting that the caspase-8/TRAF6 pathway may be the potential mechanism of TP53INP2-induced apoptosis. We hence draw a conclusion that the gene TP53INP2 could be deemed as a biomarker in prognosis and a target in ccRCC treatment.

## Figures and Tables

**Table 1:** Prime sequences.

Genes	Sequences (5′-3′)
BID-F	CATGGACCGTAGCATCCCTC
BID-R	AGCACCAGCATGGTCTTCTC
CASP4-F	AGGGCATTTGCTACCAGACC
CASP4-R	GGCAGTTGCGGTTGTTGAAT
ZFYVE1-F	AGAGAGGCGAGGAAGTCAGT
ZFYVE1-R	CCAGTACTGCCGACCAGAC
PRKAR1A-F	CACTGCTCGACCTGAGAGAC
PRKAR1A-R	GTCTGTACGAGTGCCTGCTT
NPC1-F	TGGAGGGATTGTGGTGTTGG
NPC1-R	ATCGCTCTTCAGTGGCACAA
TP53INP2-F	CACCGCTCTGGTTCTTGGACCGGCG
TP53INP2-R	AAACCGCCGGTCCAAGAACCAGAGC
HSPA8-F	GCAATGAACCCCACCAACAC
HSPA8-R	CTACTTGGACCTTGGGCCTG
EIF2S1-F	TAATAGGCGCTTGACCCCAC
EIF2S1-R	TGTTCTCTCCAGGGTTGTCG
BAG1-F	ACCCACAGCAATGAGAAGCA
BAG1-R	GTGCTGACAACGGTGTTTCC
*β*-Actin-F	GATGCTCCCCGGGCTGTATT
*β*-Actin-R	GGGGTA CTTCAGGGTCAGGA

**Table 2:** OS-related ARGs in the univariate Cox regression analysis.

Gene id	HR	HR.95L	HR.95H	*P* value	Gene id	HR	HR.95L	HR.95H	*P* value
BID	3.487	2.416	5.034	< 0.001	MAPK3	0.573	0.415	0.793	0.001
DDIT3	1.364	1.158	1.606	< 0.001	FOXO1	0.594	0.457	0.772	< 0.001
CASP3	2.013	1.380	2.935	< 0.001	CANX	0.573	0.455	0.722	< 0.001
BIRC5	1.766	1.497	2.083	< 0.001	VAMP3	0.366	0.268	0.500	< 0.001
EIF4EBP1	1.489	1.290	1.718	< 0.001	ST13	0.416	0.316	0.546	< 0.001
RGS19	1.664	1.266	2.187	< 0.001	EEF2	0.654	0.517	0.826	< 0.001
ATG4C	0.439	0.300	0.643	< 0.001	IFNG	1.432	1.176	1.743	< 0.001
MAPK8IP1	0.631	0.505	0.789	< 0.001	PIK3C3	0.454	0.305	0.677	< 0.001
DNAJB9	0.654	0.543	0.787	< 0.001	RB1	0.640	0.514	0.797	< 0.001
ATG4B	2.601	1.819	3.720	< 0.001	BCL2	0.626	0.534	0.733	< 0.001
NCKAP1	0.558	0.415	0.749	< 0.001	NBR1	0.534	0.416	0.686	< 0.001
UVRAG	0.473	0.333	0.671	< 0.001	MAPK1	0.555	0.436	0.705	< 0.001
PIK3R4	0.525	0.393	0.701	< 0.001	SIRT1	0.530	0.401	0.701	< 0.001
CASP4	2.408	1.722	3.367	< 0.001	USP10	0.516	0.353	0.754	0.001
ATF6	0.594	0.439	0.804	0.001	CHMP2B	0.586	0.430	0.799	0.001
CX3CL1	0.638	0.549	0.740	< 0.001	PTK6	1.402	1.187	1.657	< 0.001
ZFYVE1	0.429	0.298	0.618	< 0.001	TUSC1	0.577	0.450	0.739	< 0.001
PRKAR1A	0.594	0.449	0.786	< 0.001	IKBKE	1.960	1.573	2.443	< 0.001
HGS	3.008	1.953	4.632	< 0.001	BAK1	1.775	1.292	2.439	< 0.001
SH3GLB1	0.568	0.421	0.767	< 0.001	HSPA8	0.694	0.580	0.832	< 0.001
CAPN10	2.238	1.565	3.199	< 0.001	EIF2S1	0.557	0.398	0.781	0.001
ERBB2	0.651	0.531	0.800	< 0.001	CD46	0.594	0.461	0.766	< 0.001
IRGM	34.283	7.959	147.668	< 0.001	CDKN2A	1.545	1.252	1.907	< 0.001
WDFY3	0.544	0.424	0.697	< 0.001	APOL1	1.214	1.092	1.350	< 0.001
NFKB1	0.601	0.472	0.765	< 0.001	P4HB	1.490	1.175	1.889	0.001
NAF1	0.395	0.261	0.597	< 0.001	CALCOCO2	0.505	0.356	0.717	< 0.001
RAB11A	0.339	0.210	0.546	< 0.001	PINK1	0.476	0.392	0.578	< 0.001
MAPK8	0.446	0.292	0.680	< 0.001	WDR45	2.406	1.626	3.562	< 0.001
NPC1	1.552	1.199	2.010	0.001	ATG4A	0.462	0.308	0.692	< 0.001
TP53INP2	0.473	0.367	0.609	< 0.001	SPHK1	1.648	1.390	1.953	< 0.001
ATG16L2	1.388	1.161	1.659	< 0.001	PTEN	0.557	0.402	0.772	< 0.001
ULK1	1.618	1.254	2.088	< 0.001	BAG1	0.441	0.322	0.605	< 0.001
ITGA6	0.571	0.489	0.667	< 0.001	BNIP3	0.704	0.594	0.835	< 0.001
DLC1	0.713	0.587	0.865	0.001	RAB33B	0.478	0.333	0.686	< 0.001

**Table 3:** The multivariate Cox regression analysis for ARGs of overall survival.

Gene id	Coef	HR	HR.95L	HR.95H	*P* value
BID	0.632	1.882	1.185	2.988	0.007
ATG4B	1.234	3.434	1.723	6.843	< 0.001
CASP4	0.368	1.445	0.936	2.230	0.097
ZFYVE1	-0.453	0.636	0.369	1.094	0.102
PRKAR1A	0.911	2.486	1.473	4.195	0.001
CAPN10	-0.753	0.471	0.220	1.009	0.053
NFKB1	-0.720	0.487	0.304	0.780	0.003
NPC1	0.395	1.484	1.121	1.964	0.006
TP53INP2	-0.477	0.621	0.429	0.897	0.011
ULK1	0.368	1.444	1.028	2.030	0.034
MAPK1	0.470	1.600	0.949	2.698	0.078
HSPA8	-0.500	0.606	0.442	0.833	0.002
EIF2S1	0.883	2.418	1.414	4.135	0.001
CDKN2A	-0.228	0.796	0.619	1.024	0.076
PTEN	-0.722	0.486	0.308	0.766	0.002
BAG1	-0.611	0.543	0.365	0.806	0.002
BNIP3	-0.359	0.698	0.558	0.874	0.002

**Table 4:** The univariate Cox analysis of clinicopathological parameters and prognosis-related prediction model with the OS in ccRCC patients.

Gene id	HR	HR.95L	HR.95H	*P* value
Age	1.033	1.019	1.047	< 0.001
Gender	0.931	0.675	1.284	0.663
Grade	2.293	1.854	2.836	< 0.001
AJCC stage	1.889	1.649	2.164	< 0.001
T	1.941	1.639	2.299	< 0.001
M	4.284	3.106	5.908	< 0.001
Risk score	1.220	1.181	1.261	< 0.001

## Data Availability

The data used to support the findings of this study are included within the article.

## Supplementary Materials

Supplementary 1 Supplementary Figure 1: GO enrichment analysis and KEGG analysis on the autophagy-related genes (ARGs) of differential expression. (A) GO enrichment analysis on the ARGs of differential expression. The top 10 DEG-associated cellular components, biological functions, and molecular functions. The barplot and circle of KEGG enrichment analyses. (B) The KEGG analysis on the ARGs of differential expression. (C) The outer circle represented a scatter plot for each term of the logFC of differentially expressed ARGs. Red circles showed upregulations, whereas the blue ones displayed downregulations. The high Z-score value revealed a higher expression in the enriched pathway. (D) Heatmap of the relationship amid ARGs and KEGG enrichment. The color of each block was closely related with the logFC values.

Supplementary 2 Supplementary Figure 2: the high expression of BID, ATG4B, CASP4, CAPN10, and ULK1 was greatly correlated with worse overall survival in Kaplan-Meier curves.

Supplementary 3 Supplementary Figure 3: the low expression of ZFYVE1, PRKAR1A, NFKB1, TP53INP2, MAPK1, HSPA8, BAG1, EIF2S1, PTEN, and BNIP3 was notably related to worse overall survival observed in Kaplan-Meier curves.

Supplementary 4 Supplementary Figure 4: the expression level of NPC1 and CDKN2A had no significant correlation with OS in Kaplan-Meier curves.
