# Supplementary figures and images for: Overexpression of TP53INP2 Promotes Apoptosis in Clear Cell Renal Cell Cancer via Caspase-8/TRAF6 Signaling Pathway
Source: J Immunol Res. 2022 May 14;2022:1260423. doi: 10.1155/2022/1260423 (PMC9125430; doi:10.1155/2022/1260423)

A

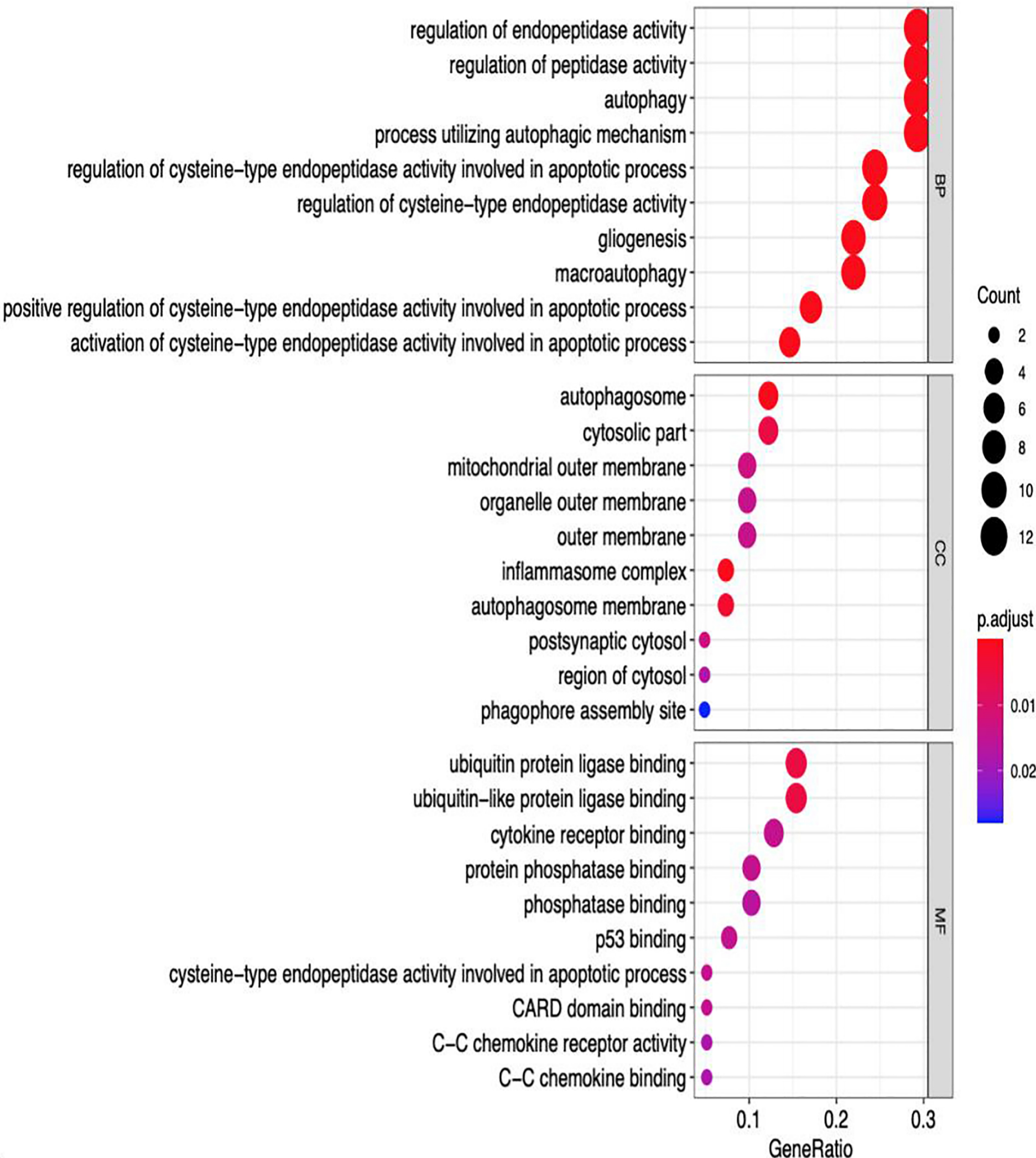

B

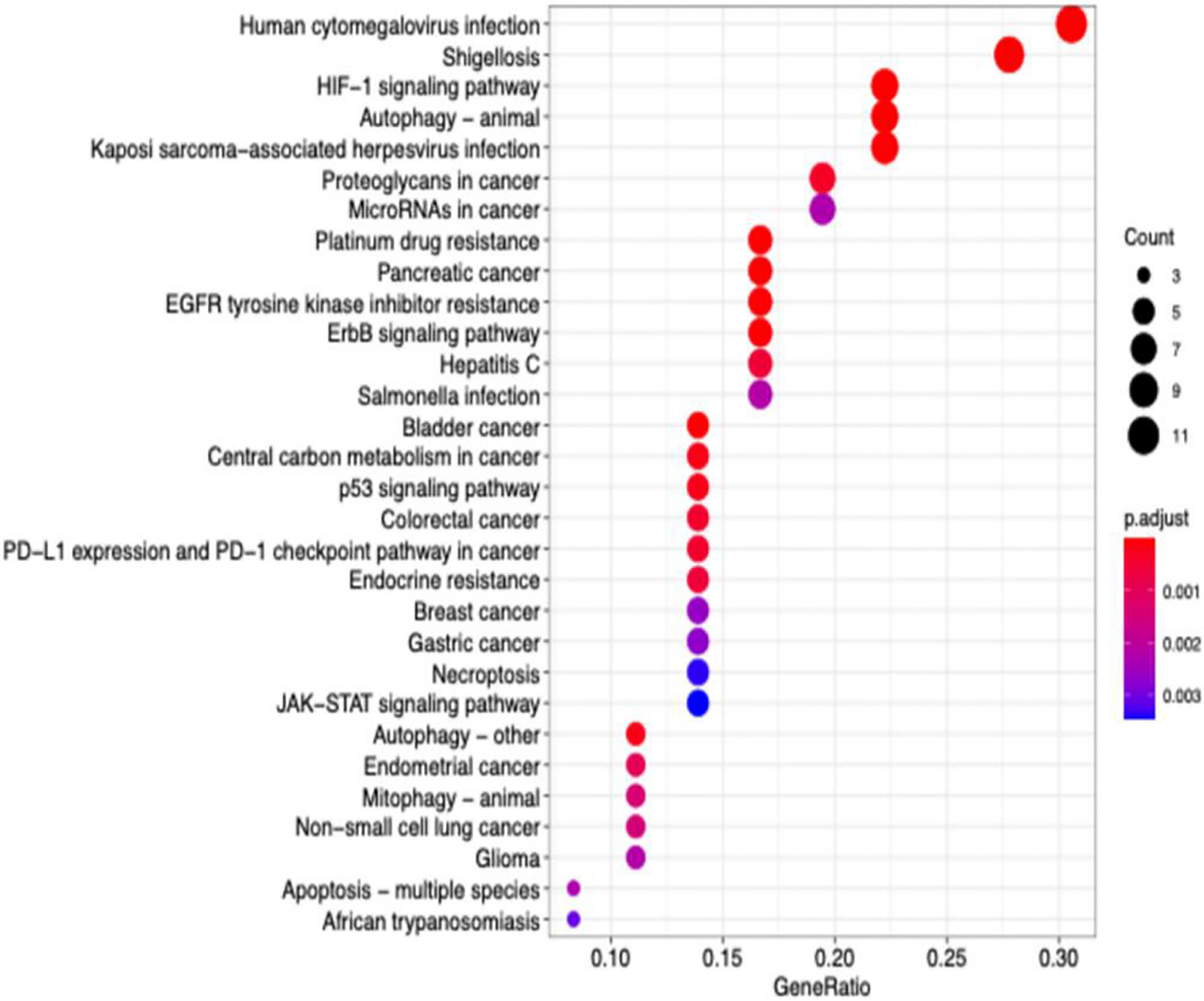

C

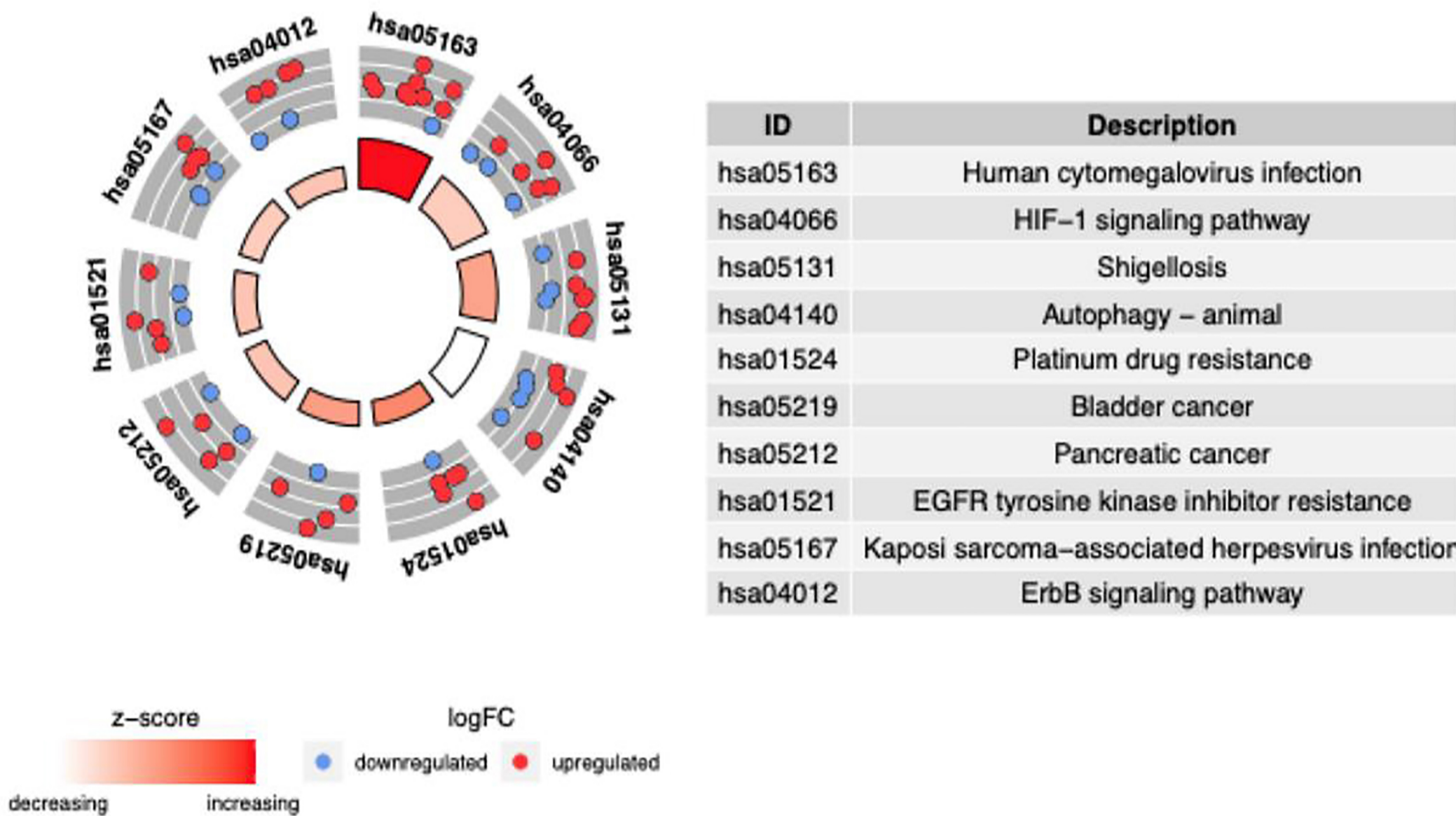

D

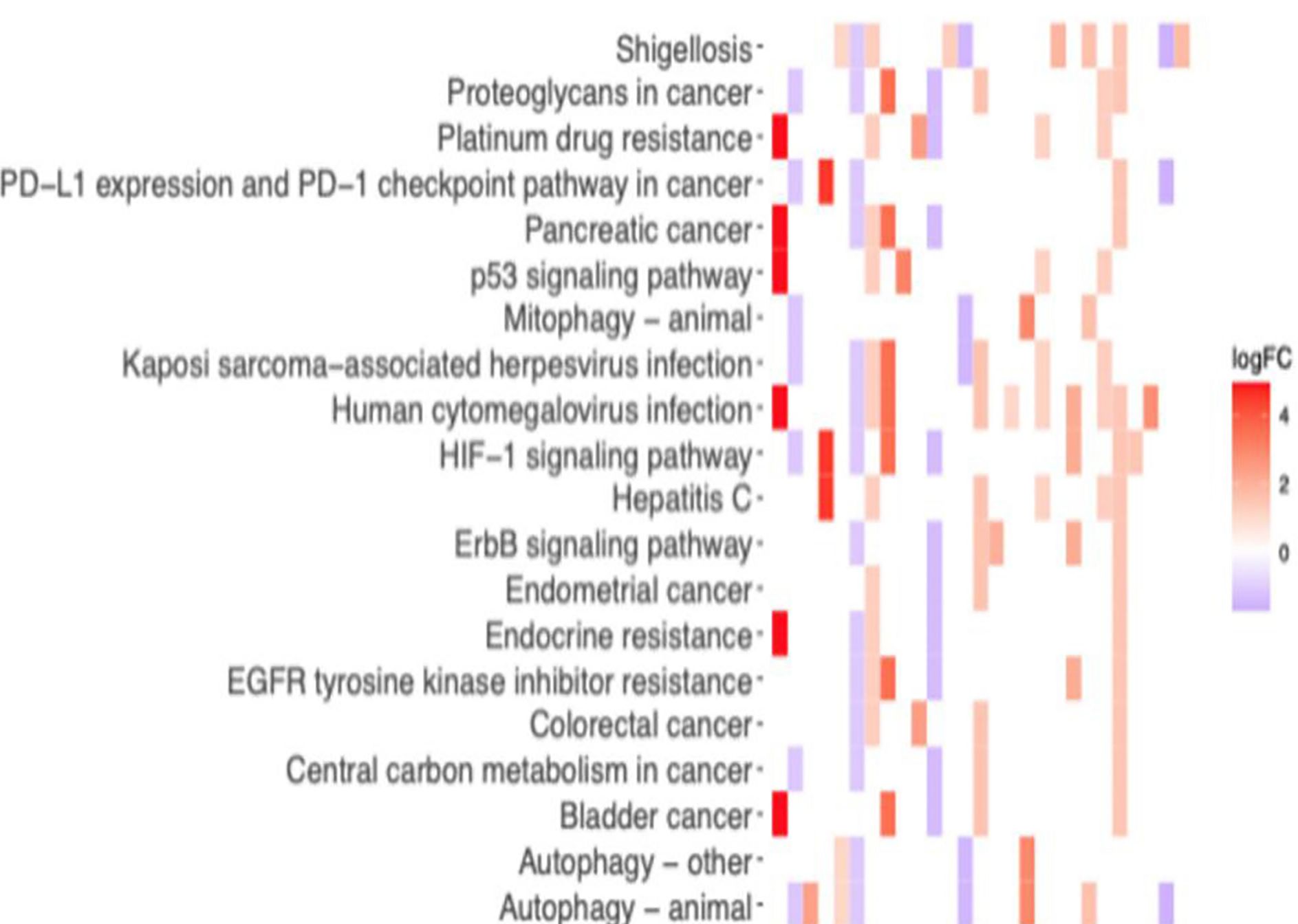

Supplement: Supplementary 1 — Supplementary Figure 1: GO enrichment analysis and KEGG analysis on the autophagy-related genes (ARGs) of differential expression. (A) GO enrichment analysis on the ARGs of differential expression. The top 10 DEG-associated cellular components, biological functions, and molecular functions. The barplot and circle of KEGG enrichment analyses. (B) The KEGG analysis on the ARGs of differential expression. (C) The outer circle represented a scatter plot for each term of the logFC of differentially expressed ARGs. Red circles showed upregulations, whereas the blue ones displayed downregulations. The high Z-score value revealed a higher expression in the enriched pathway. (D) Heatmap of the relationship amid ARGs and KEGG enrichment. The color of each block was closely related with the logFC values. [file 1260423.f1.pdf]

**BID (p<0.001)**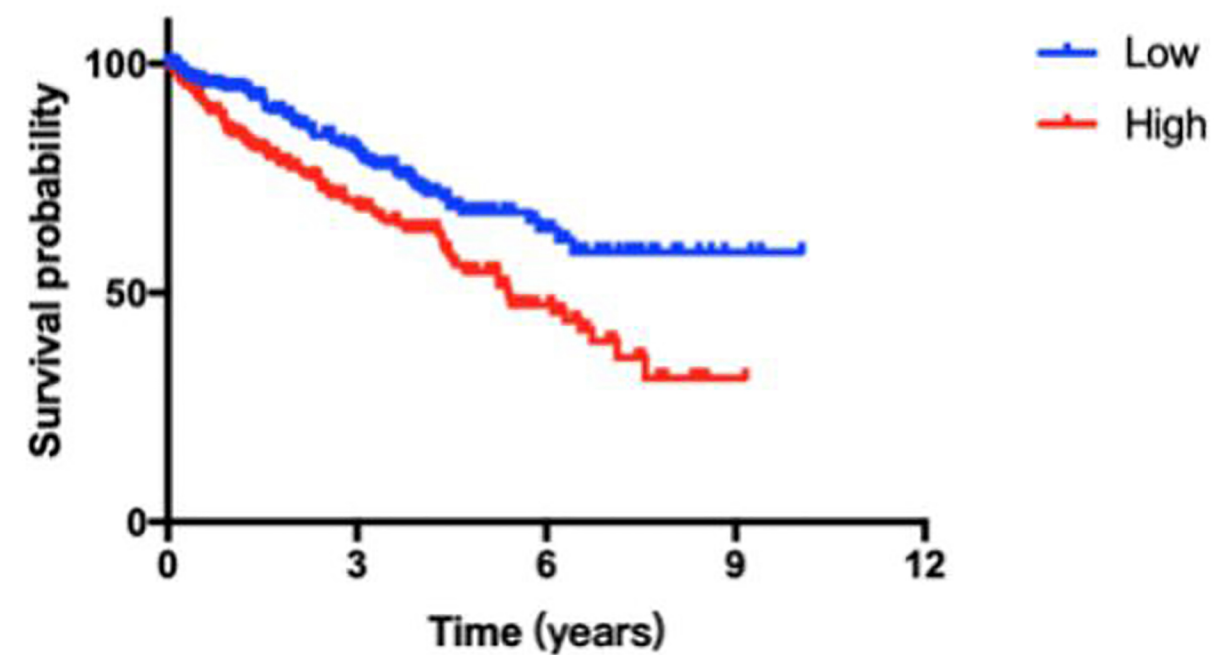**ATG4B (p<0.001)**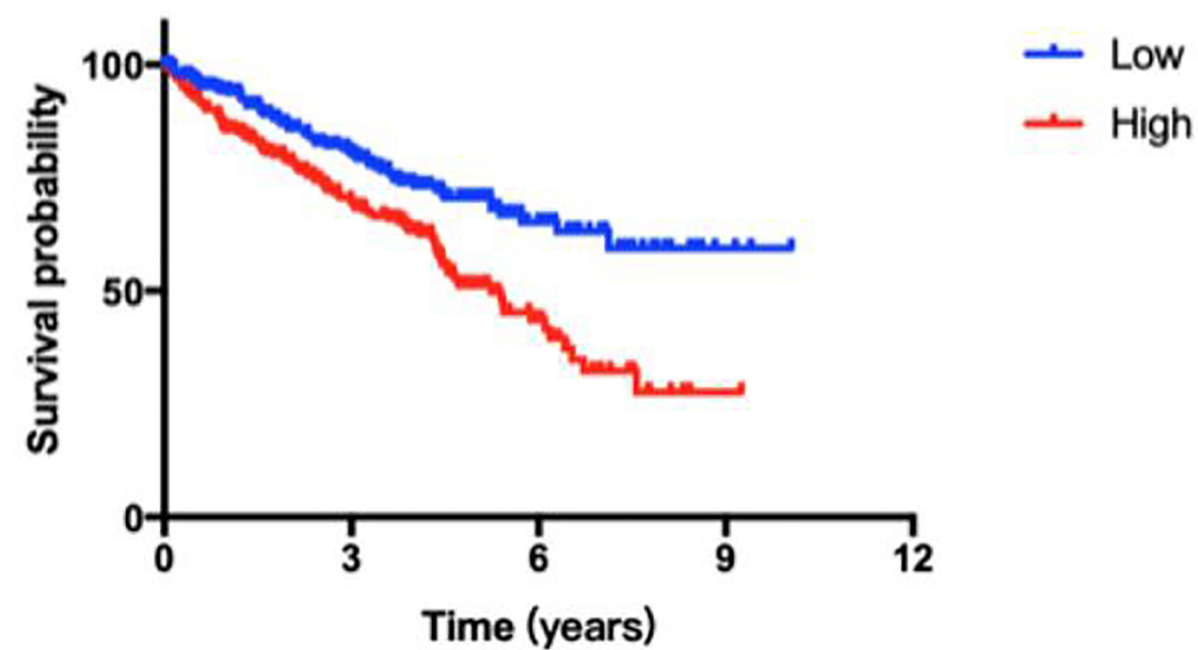**CASP4 (p=0.001)**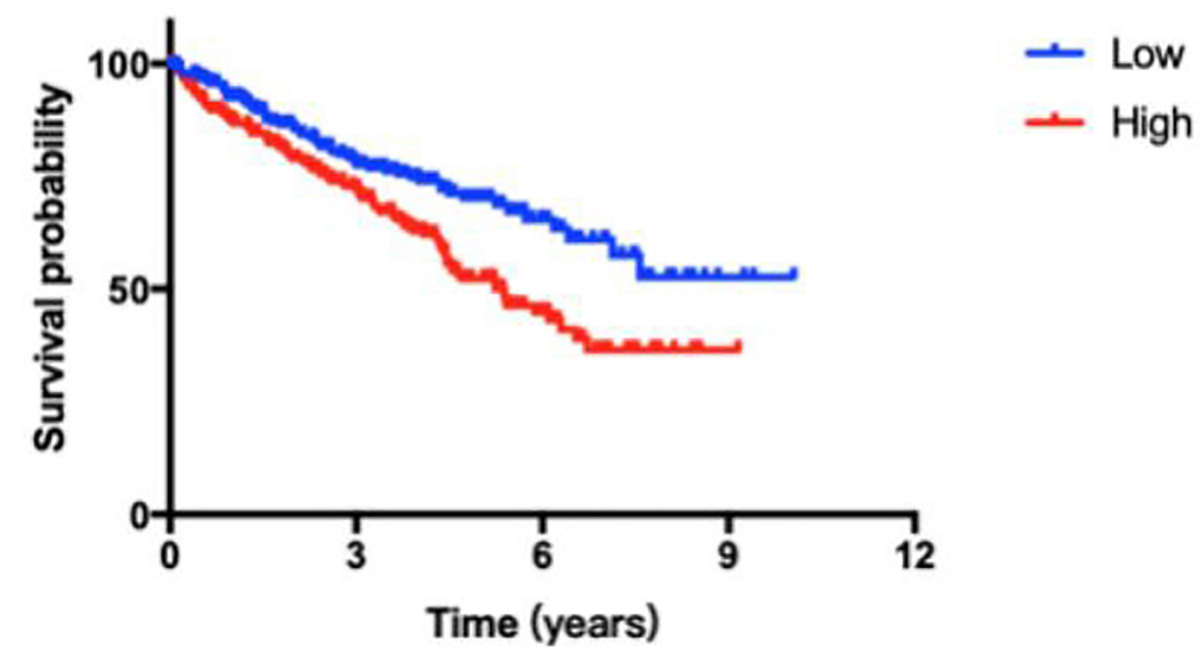**CAPN10 (p=0.021)**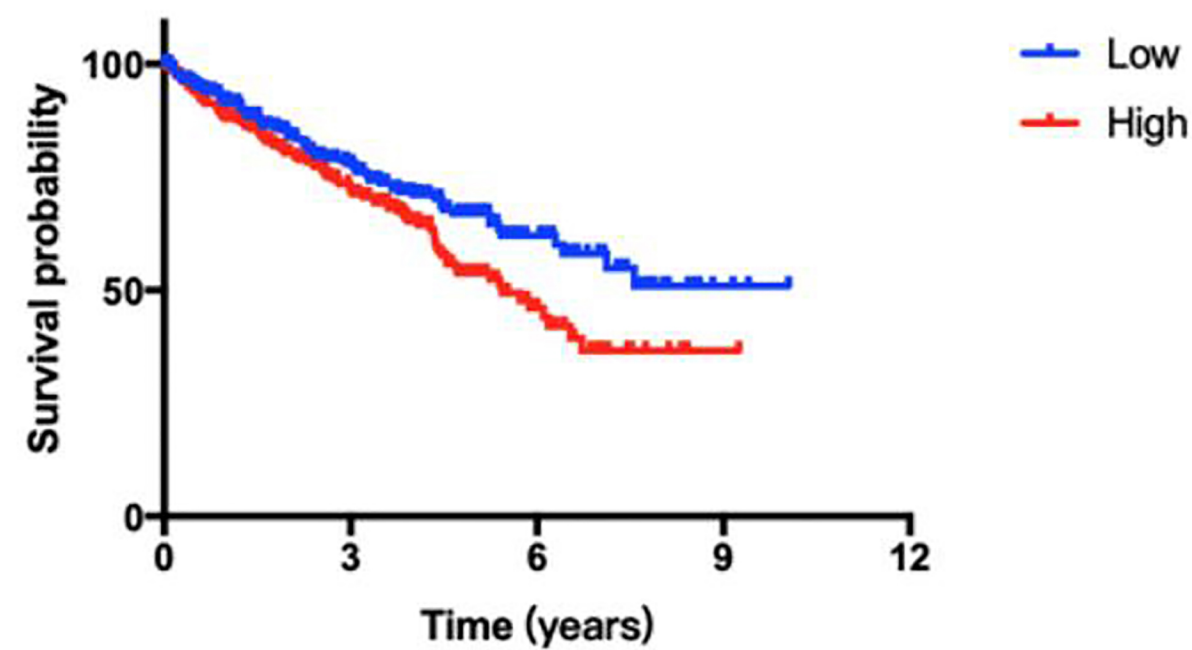**ULK1 (p<0.001)**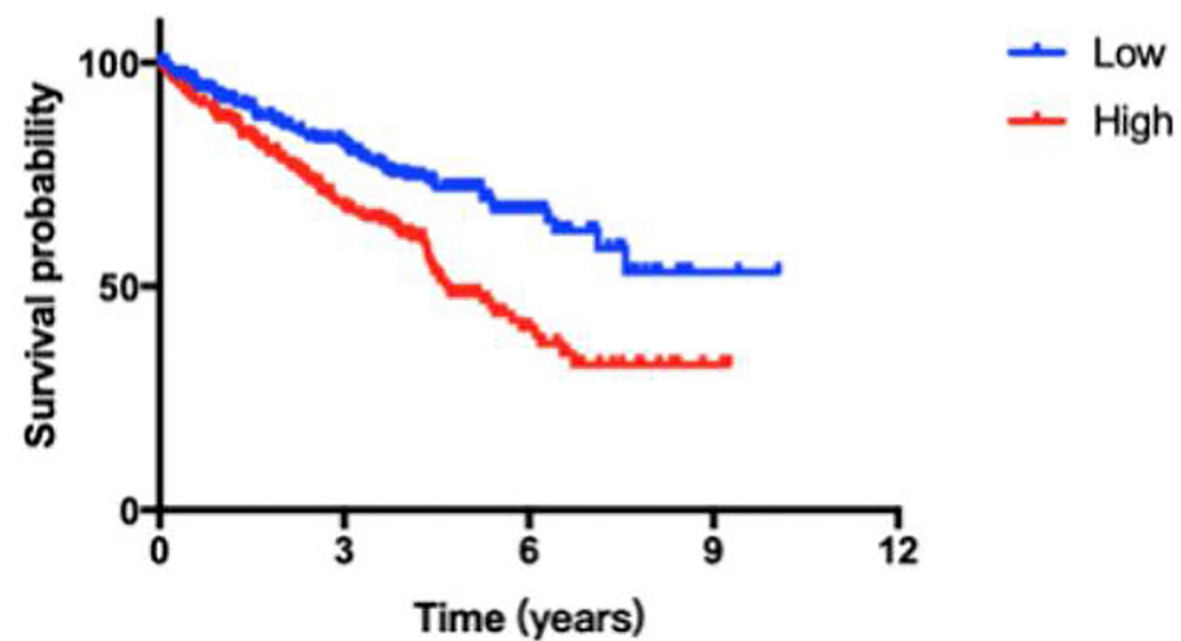

Supplement: Supplementary 2 — Supplementary Figure 2: the high expression of BID, ATG4B, CASP4, CAPN10, and ULK1 was greatly correlated with worse overall survival in Kaplan-Meier curves. [file 1260423.f2.pdf]

**ZFYVE1 (p<0.001)**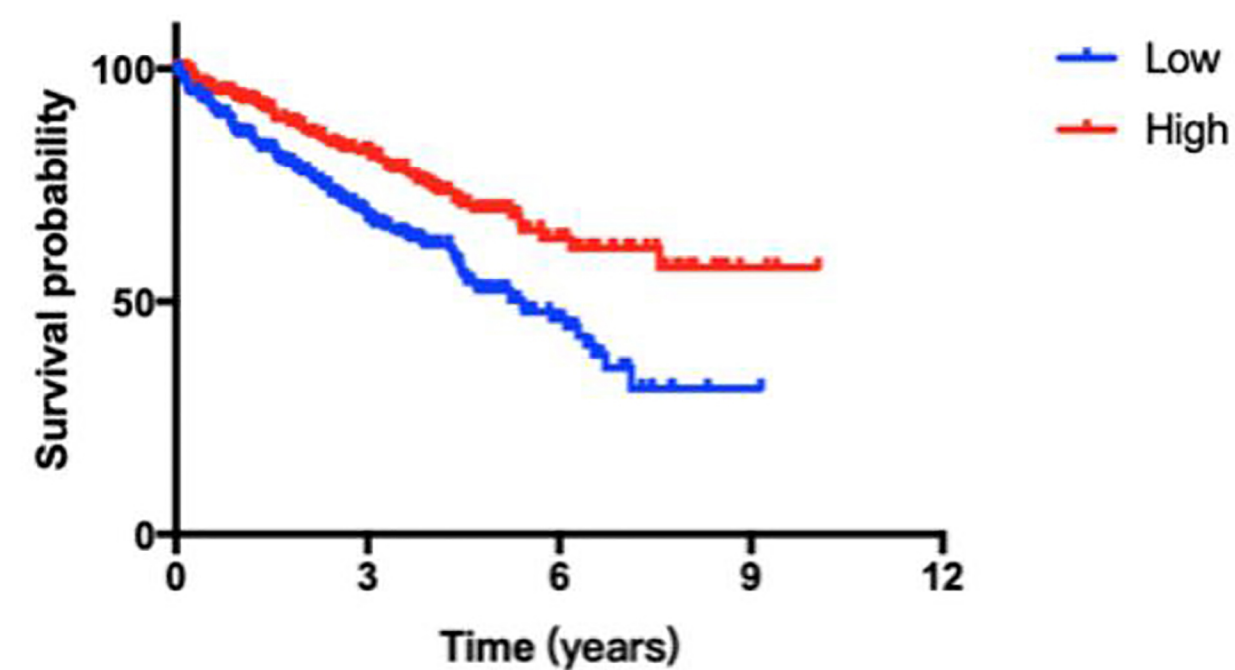**PRKAR1A (p<0.001)**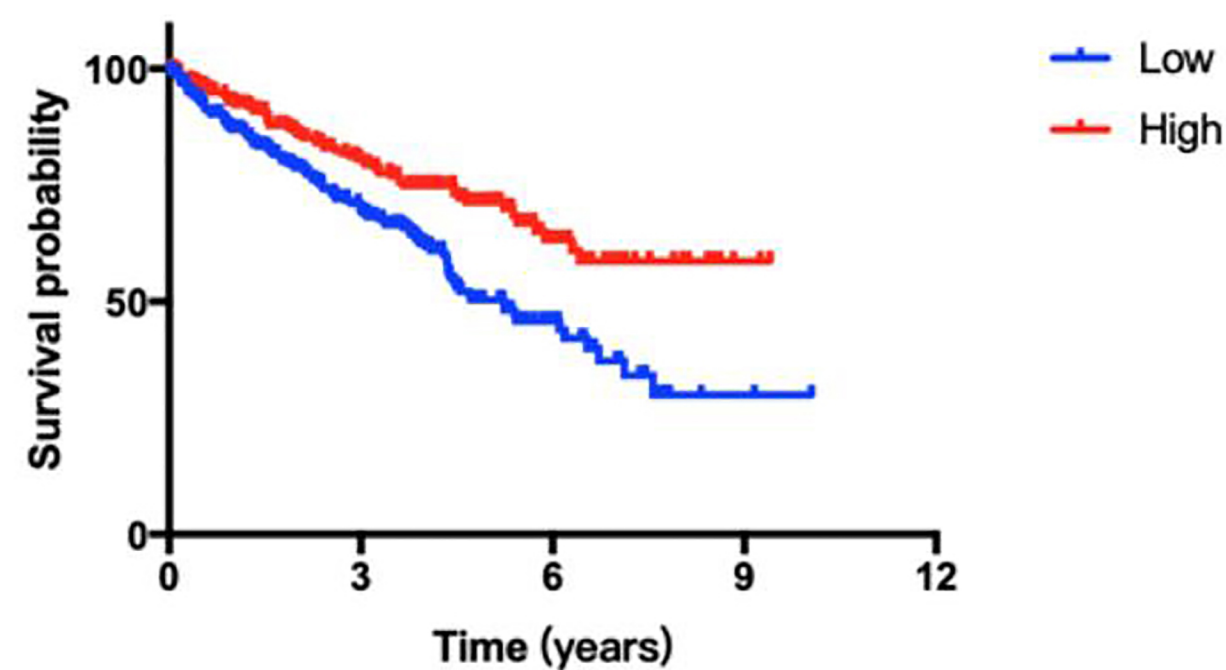**NFKB1 (p<0.001)**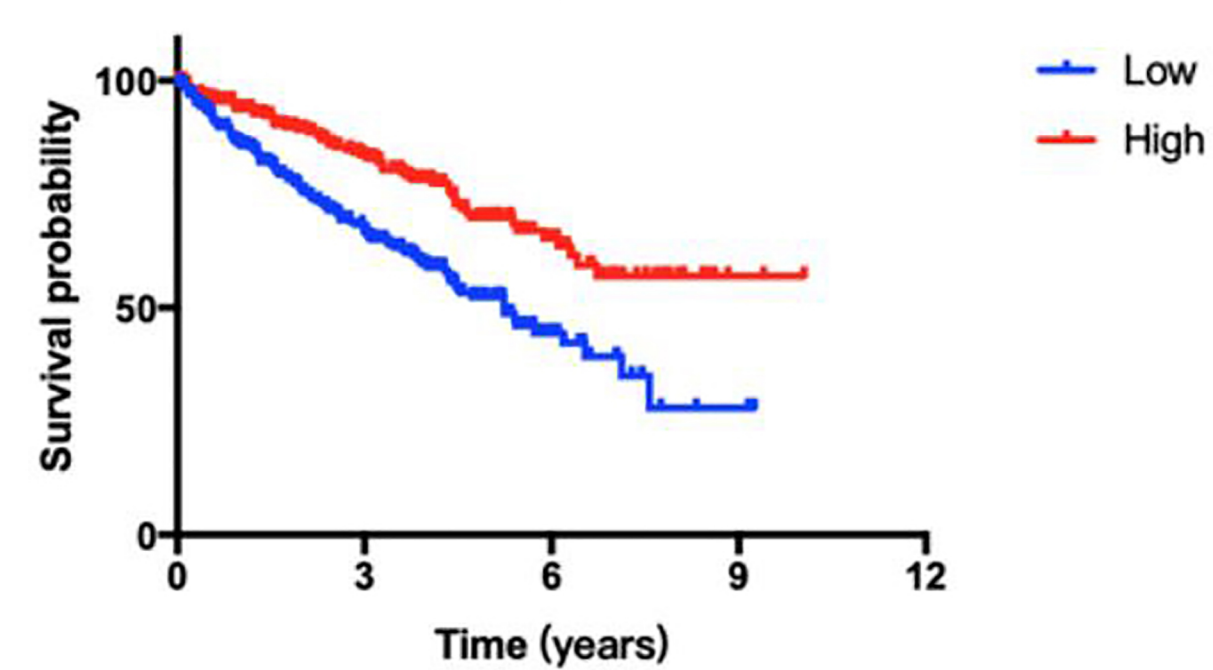**TP53INP2 (p<0.001)**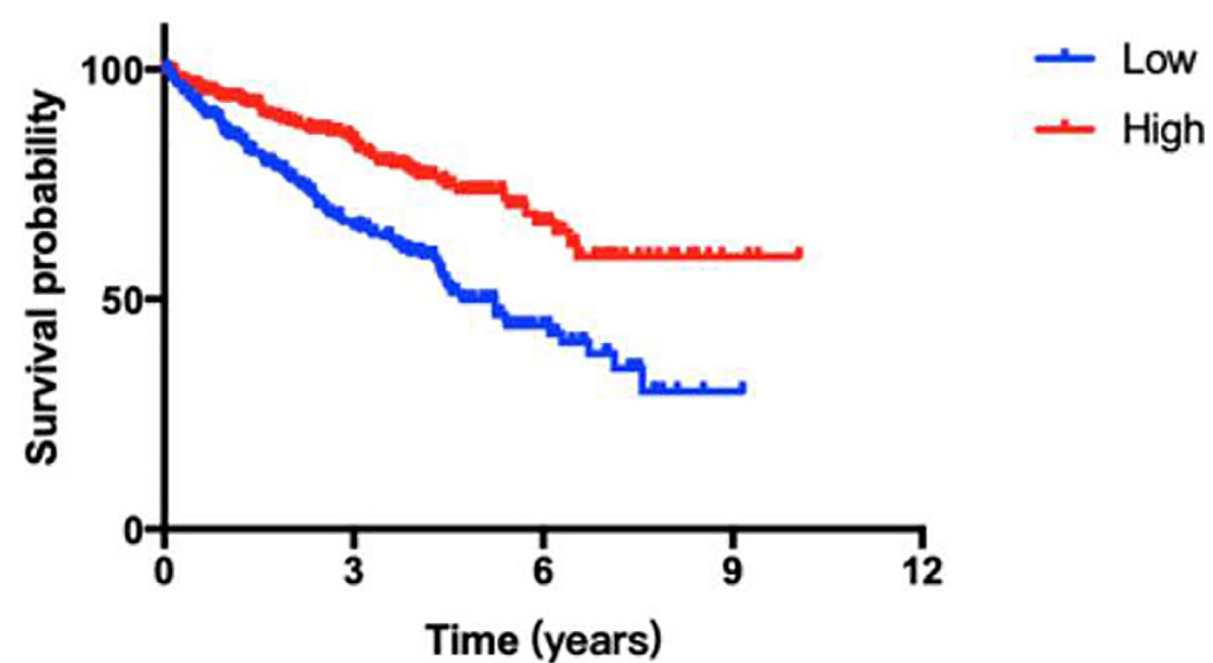**MAPK1 (p<0.001)**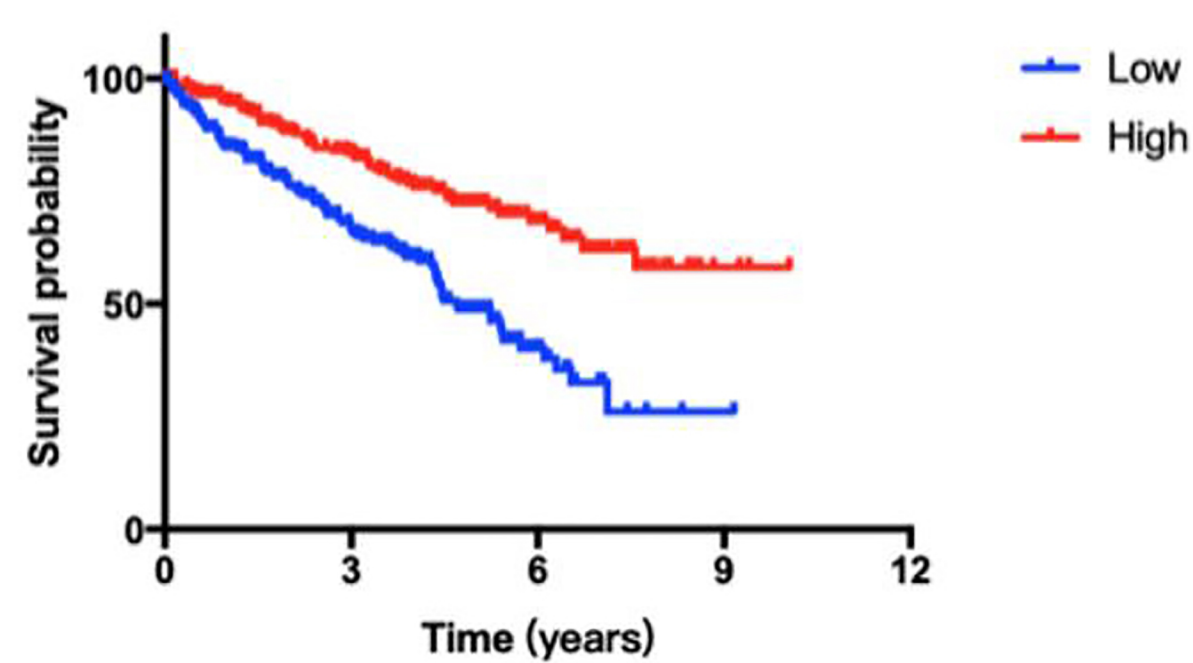**HSPA8 (p=0.036)**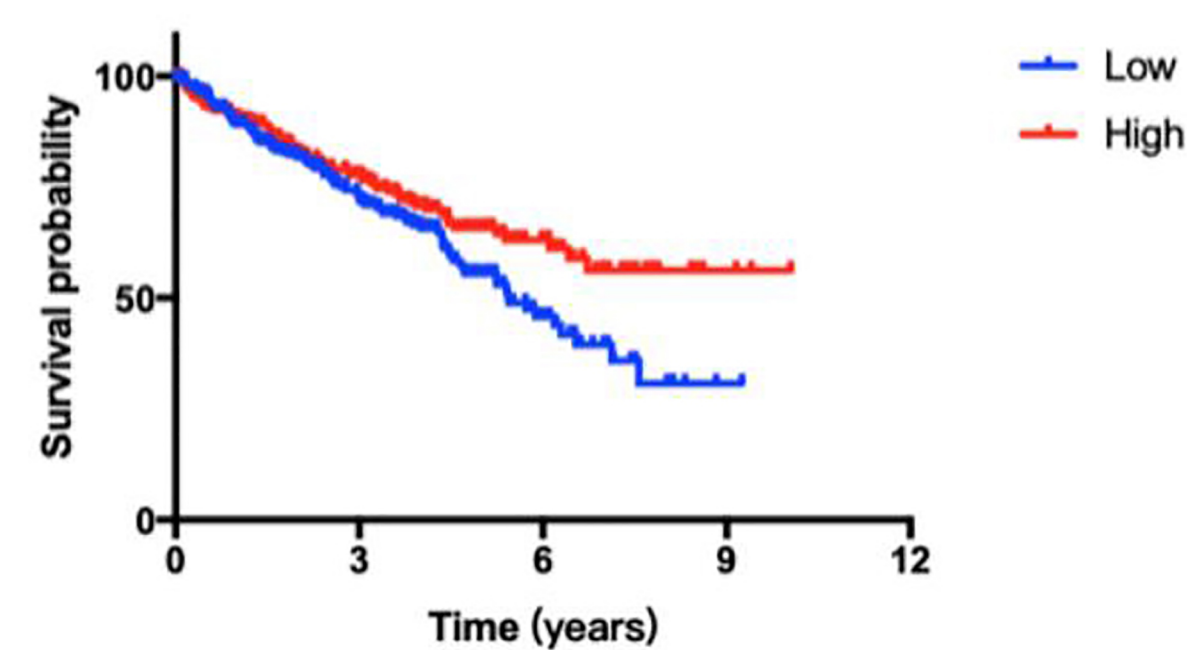**BAG1 (p<0.001)**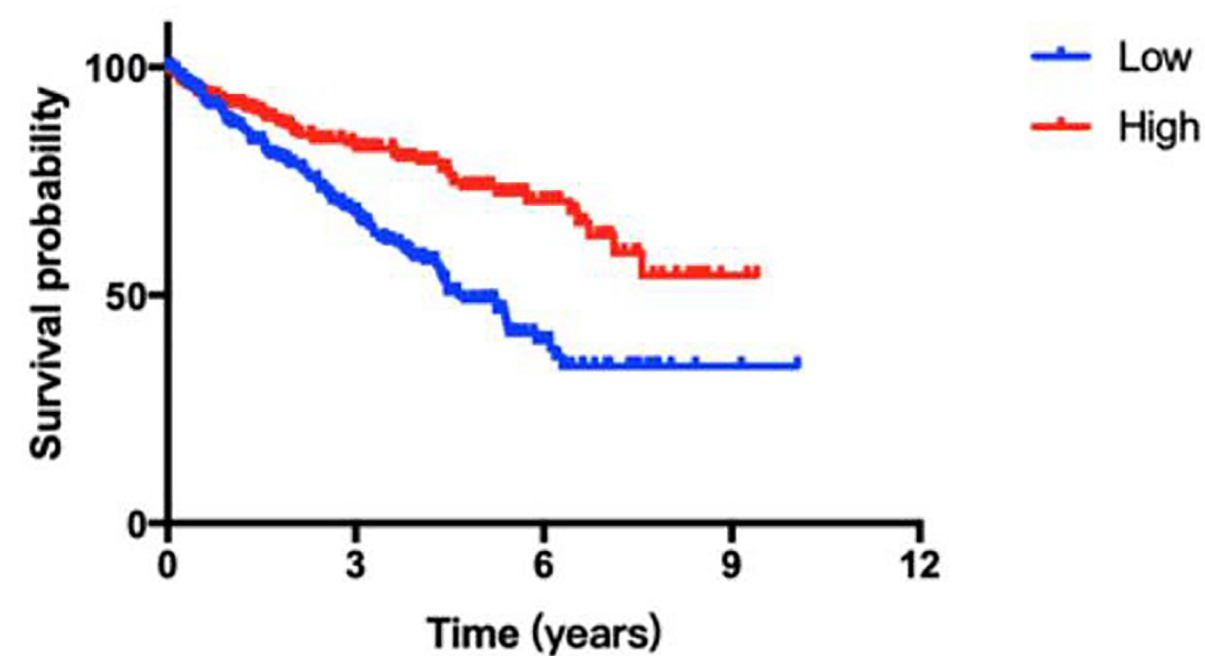**EIF2S1 (p<0.001)**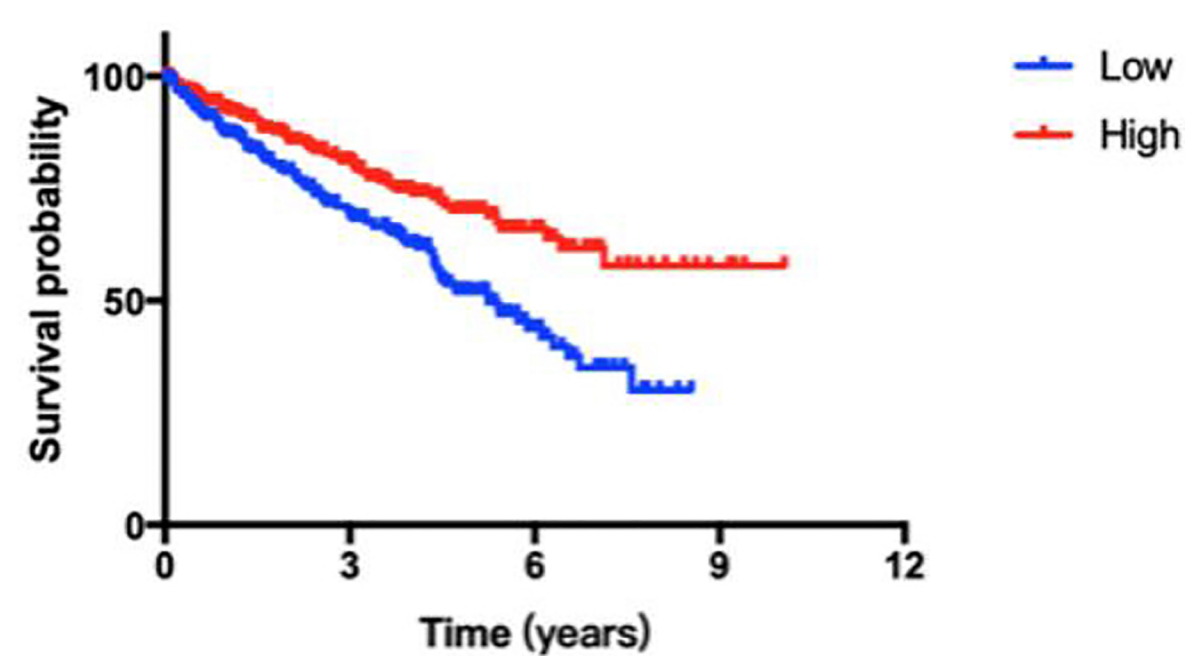**PTEN (p<0.001)**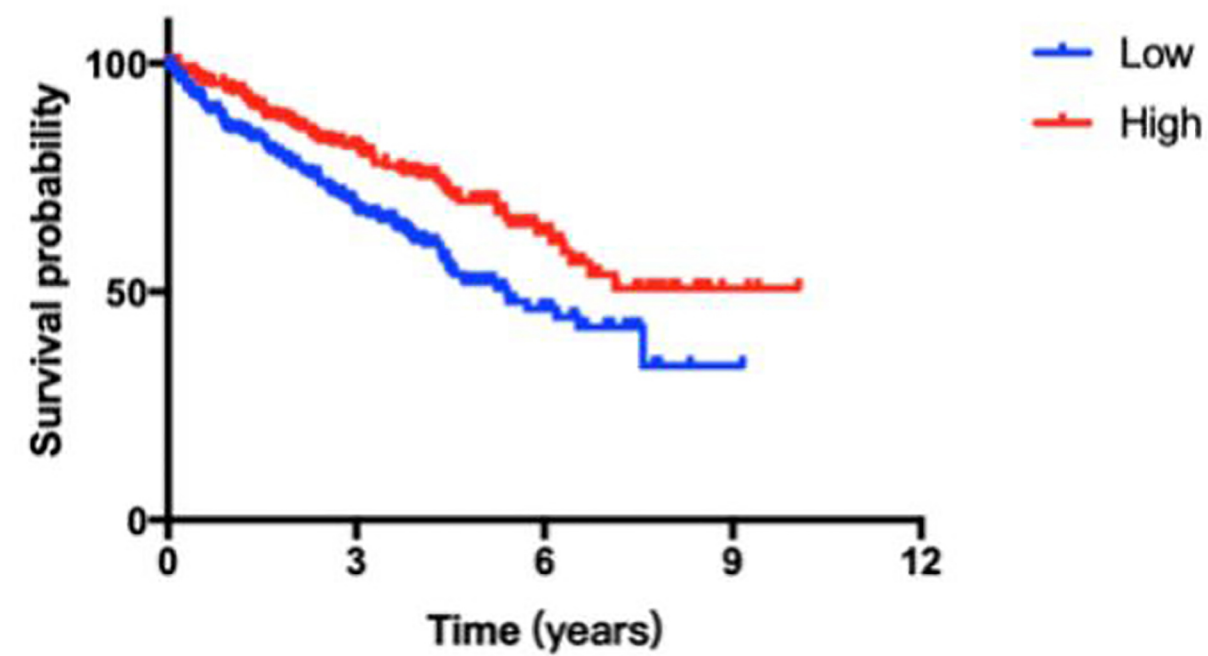**BNIP3 (p=0.003)**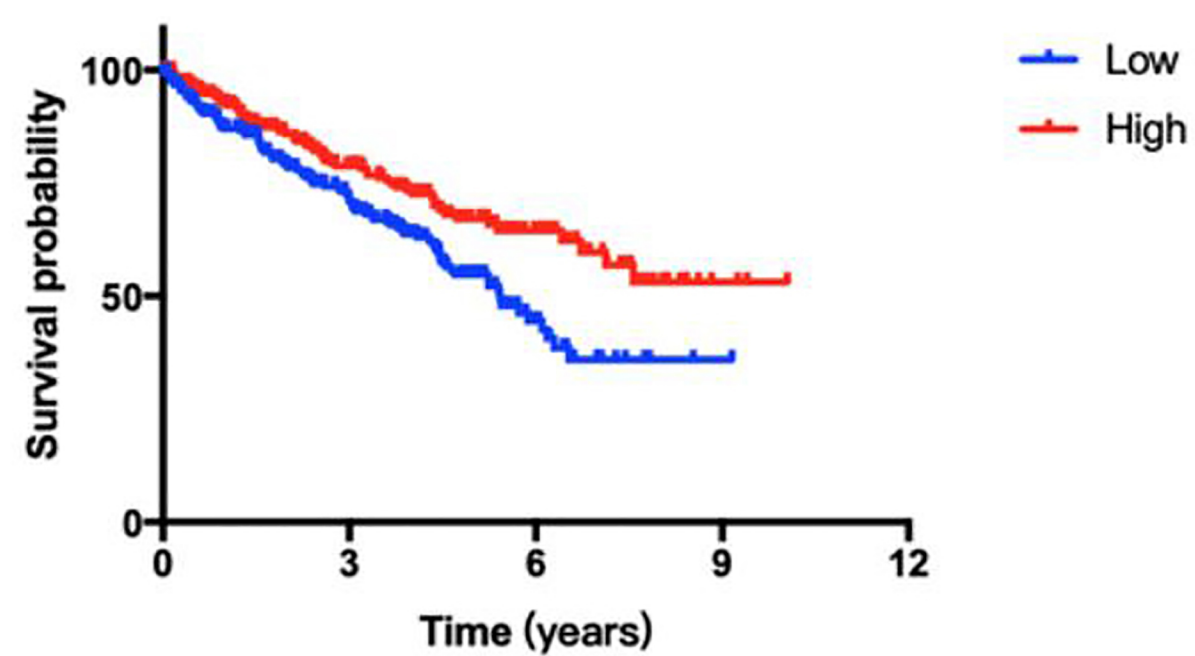

Supplement: Supplementary 3 — Supplementary Figure 3: the low expression of ZFYVE1, PRKAR1A, NFKB1, TP53INP2, MAPK1, HSPA8, BAG1, EIF2S1, PTEN, and BNIP3 was notably related to worse overall survival observed in Kaplan-Meier curves. [file 1260423.f3.pdf]

**NPC1 (p=0.207)**

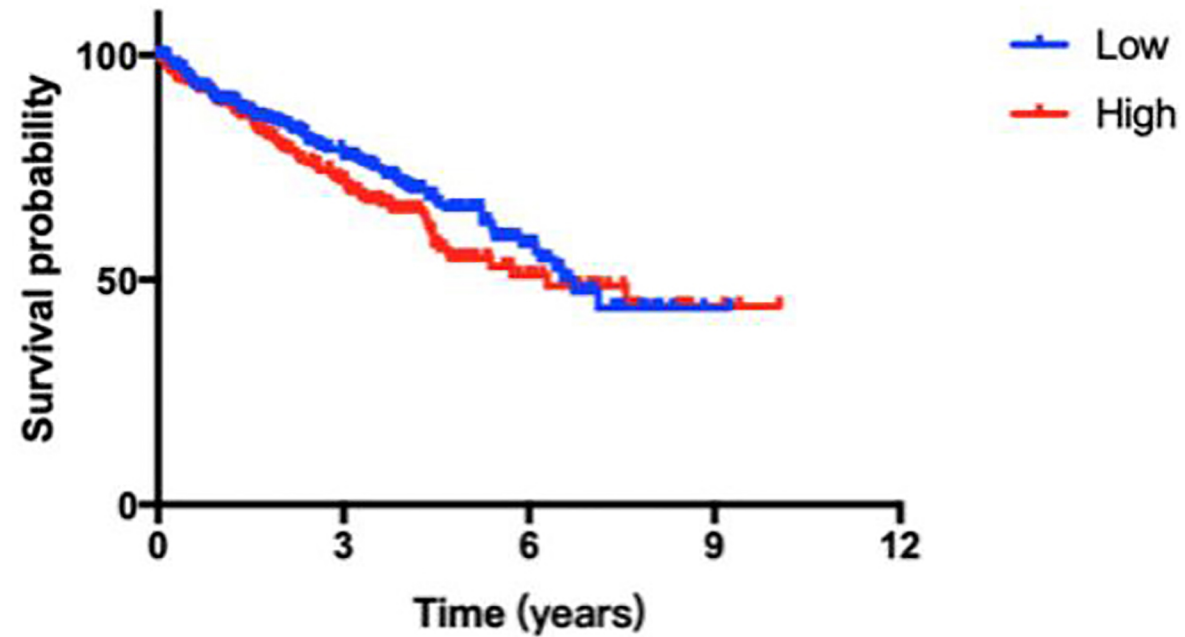

**CDKN2A (p=0.053)**

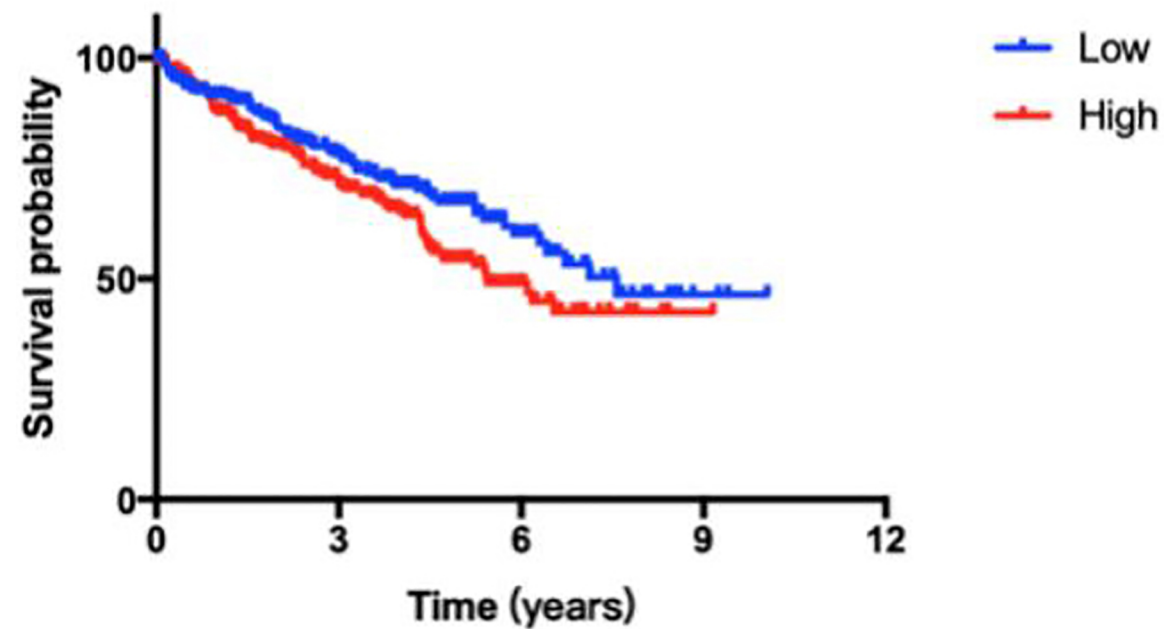

Supplement: Supplementary 4 — Supplementary Figure 4: the expression level of NPC1 and CDKN2A had no significant correlation with OS in Kaplan-Meier curves. [file 1260423.f4.pdf]
